# Regulation of Ascorbate-Glutathione Pathway in Mitigating Oxidative Damage in Plants under Abiotic Stress

**DOI:** 10.3390/antiox8090384

**Published:** 2019-09-09

**Authors:** Mirza Hasanuzzaman, M. H. M. Borhannuddin Bhuyan, Taufika Islam Anee, Khursheda Parvin, Kamrun Nahar, Jubayer Al Mahmud, Masayuki Fujita

**Affiliations:** 1 Department of Agronomy, Faculty of Agriculture, Sher-e-Bangla Agricultural University, Dhaka 1207, Bangladesh; 2 Laboratory of Plant Stress Responses, Department of Applied Biological Science, Faculty of Agriculture, Kagawa University, Miki-cho, Kita-gun, Kagawa 761-0795, Japan (M.H.M.B.B.) (K.P.); 3 Citrus Research Station, Bangladesh Agricultural Research Institute, Jaintapur, Sylhet 3156, Bangladesh; 4 Department of Horticulture, Faculty of Agriculture, Sher-e-Bangla Agricultural University, Dhaka 1207, Bangladesh; 5 Department of Agricultural Botany, Faculty of Agriculture, Sher-e-Bangla Agricultural University, Dhaka 1207, Bangladesh; 6 Department of Agroforestry and Environmental Science, Faculty of Agriculture, Sher-e-Bangla Agricultural University, Dhaka 1207, Bangladesh

**Keywords:** antioxidant defense, free radicals, glyoxalase system, hydrogen peroxide, plant abiotic stress, reactive oxygen species, redox biology, stress signaling

## Abstract

Reactive oxygen species (ROS) generation is a usual phenomenon in a plant both under a normal and stressed condition. However, under unfavorable or adverse conditions, ROS production exceeds the capacity of the antioxidant defense system. Both non-enzymatic and enzymatic components of the antioxidant defense system either detoxify or scavenge ROS and mitigate their deleterious effects. The Ascorbate-Glutathione (AsA-GSH) pathway, also known as Asada–Halliwell pathway comprises of AsA, GSH, and four enzymes viz. ascorbate peroxidase, monodehydroascorbate reductase, dehydroascorbate reductase, and glutathione reductase, play a vital role in detoxifying ROS. Apart from ROS detoxification, they also interact with other defense systems in plants and protect the plants from various abiotic stress-induced damages. Several plant studies revealed that the upregulation or overexpression of AsA-GSH pathway enzymes and the enhancement of the AsA and GSH levels conferred plants better tolerance to abiotic stresses by reducing the ROS. In this review, we summarize the recent progress of the research on AsA-GSH pathway in terms of oxidative stress tolerance in plants. We also focus on the defense mechanisms as well as molecular interactions.

## 1. Introduction

With the advancement of lifestyle the natural resources are being exploited and the interruption of natural environment is increasing the extremity of various kinds of abiotic stress, including salt stress, drought stress, waterlogging, temperature extremes, including high and low, excess and low light intensity, radiation stress, ozone, metal and metalloid toxicity, and other organic or inorganic pollutants. Environmental extremity narrows ways to increase plant productivity. Ever-increasing population demands newly cultivable areas, including the adverse land areas, even the higher crop production in per unit area. 

Any abiotic stress impaired stomatal function, photosystem activity, Calvin cycle, or photosynthetic enzyme activities, as well as altered electron transport chain reactions. Moreover, unfavorable peroxisomal or cytosolic atmosphere led to overwhelm of electron absorption and generate ROS as a common outcome and subsequently causes oxidative damage [1,2]. If the challenges of plant scientists are increasing productivity against the abiotic stresses, their concentrations are moving to the depth for breaking the obstacles at the cellular or organelles levels, where abiotic stresses impose common types of barrier to hinder their function. Reactive oxygen species is an inescapable outcome of aerobic reactions, which are partly reduced or activated by the appearance of oxygen. Reactive oxygen species is a combined name that indicates different, highly active components. Superoxide (O_2_^−^), hydroxyl (OH^•^), and peroxyl (ROO^•^) are some examples of oxygen radicals. Hydrogen peroxide (H_2_O_2_), singlet oxygen (^1^O_2_), and ozone (O_3_) are the non-radical types of ROS [3]. Reactive oxygen species are important for plants. They have dual role in plants: a small amount of those acts as a signal for inducing abiotic stress responses towards adaptation process, while the excess generation of those causes oxidative damage. However, in severe cases, oxidative damages to membranes (lipid peroxidation), proteins, nucleic acid, including RNA and DNA, and even directs to the oxidative obliteration of the cell [4]. Chloroplast, mitochondrion, membranes of the cell or its ultrastructural organelles, apoplast, and nucleolus are the locations of ROS production. Nonetheless, peroxisome is also considered as a powerful source of ROS since the electron transport chain (ETC) and photochemical reactions are the majority of the processes generating ROS [5,6,7]. 

Plants have an antioxidant defense system having non-enzymatic and enzymatic antioxidants in cellular organelles, which scavenges different ROS up to a certain level. If the ROS generation is higher than the scavenging ability of the antioxidant system, then oxidative damage occurs. Antioxidant defense system comprises ascorbate (AsA), glutathione (GSH), carotenoids, tocopherols, flavonoids, etc., which are some commonly known non-enzymatic antioxidants [5]. Ascorbate peroxidase (APX), monodehydroascorbate reductase (MDHAR), dehydroascorbate reductase (DHAR), glutathione reductase (GR), superoxide dismutase (SOD), catalase (CAT), glutathione peroxidase (GPX), glutathione *S*-transferase (GST), and peroxiredoxin (PRX) are well known enzymatic antioxidant components [8,9]. Among all of these, AsA, GSH, APX, MDHAR, DHAR, and GR comprise the AsA-GSH cycle.

Ascorbate is one of the most powerful substrates for scavenging H_2_O_2_. Ascorbate maintains the reduced state of α-tocopherol. Ascorbate is supposed to be concerned in zeaxanthin biosynthesis dissipating excess light energy in the thylakoid membranes of chloroplast and prevents oxidative stress. Ascorbate sustains reduce the state of prosthetic metal ions and maintain the activity of antioxidant enzymes [6]. Glutathione regulates various metabolic functions; it acts as an antioxidant. Glutathione peroxidase and GST utilize GSH as substrate; GPX is responsible for ROS detoxification, whereas GST is liable for xenobiotic detoxification [1]. The glyoxalase system consisting of glyoxalase I (Gly I) and glyoxalase II (Gly II) enzymes detoxifies cytotoxic and oxidative stress creator methylglyoxal (MG), where Gly I uses GSH and after finishing MG detoxification, GSH is recycled [2]. The positive role of AsA-GSH cycle components has been documented in many plants that are affected by abiotic stresses [1,2]. Participation of the GSH/glutathione disulfide (GSSG, the oxidized form of GSH) redox in maintaining a favorable cellular environment and in stress signal and adaptation were discussed in some previous findings. Glutathione participates in signal transduction, the proper pathway, of which remains unrevealed. The presence of AsA and GSH has been reported to improve osmoregulation, plant water status and nutrient status, water use efficiency, photosynthetic performance, and the overall productivity of plants. Exogenous AsA and GSH applications have been reported to enhance the antioxidant defense as well as the overall tolerance of plants against abiotic stresses. Accordingly, the enzymatic antioxidants of AsA-GSH cycle participates in scavenging ROS, whereas AsA and GSH not only directly scavenge a range of ROS but also perform many other functions to maintain a favorable state in cytosol and other cellular organelles to enhance antioxidant capacity and to reduce oxidative stress, which is induced by different abiotic stresses; AsA and GSH also improve the physiological performance of plants. Since the discovery of the AsA-GSH cycle, its most discussed topics are related to antioxidative protection. However, in this aspect, various other factors should be revealed like physiological factors/processes involved in generating oxidative stress, role of AsA-GSH cycle components in regulating those physiological processes and ultimately the oxidative stress. Considering the multiple vital roles of AsA-GSH cycle in mitigating oxidative stress, this review accommodates presently available and updates of research findings and perspectives.

## 2. Ascorbate-Glutathione Pathway—An Overview

**Figure 1:**
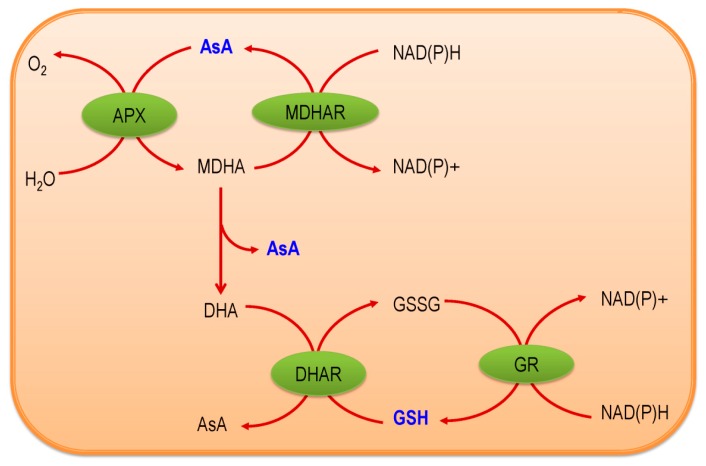
Ascorbate-Glutathione (AsA-GSH) (Ascorbate-Glutathione) pathway [ascorbate, AsA; ascorbate peroxidase, APX; monodehydroascorbate, MDHA; monodehydroascorbate reductase, MDHAR; dehydroascorbate, DHA; dehydroascorbate reductase, DHAR; glutathione, GSH; oxidized glutathione, GSSG; glutathione reductase, GR; Nicotinamide adenine dinucleotide phosphate (reduced form), NAD(P)H; Nicotinamide adenine dinucleotide phosphate (oxidized form), NAD(P)^+^].

Ascorbate-Glutathione pathway (also called as Asada–Halliwell pathway) is the major pathway of antioxidant defense, which mainly detoxify the H_2_O_2_ in a plant cell. Apart from AsA and GSH, its enzymes—APX, MDHAR, DHAR, and GR [6]—have significant roles. Both AsA and GSH are found in the cytosol, nucleus, chloroplast, mitochondria, and peroxisome, where they operate the functions assisted by four enzymes and, therefore, each enzyme has several isoforms that are based on the cellular localization [10]. Both AsA and GSH are present in cellular organelles in a millimolar range, for instance, in *Arabidopsis thaliana*, AsA concentration is the highest (22.8 mM) in the peroxisome, where GSH is highest (14.9 mM) in mitochondria [11,12]. AsA and GSH both have high redox potentials and, therefore, interact with many components and pathways towards the maintenance of a generally reduced state. There are few steps, by which AsA and GSH work coordinately to detoxify H_2_O_2,_ and at the same time, both AsA and GSH are regenerated. First, the enzyme APX converts H_2_O_2_ into the water with the help of AsA as an electron donor, which is also converted into monodehydroascorbate (MDHA). This MDHA again regenerates AsA by the activity of MDHAR and a part of this is spontaneously converted into dehydroascorbate (DHA). Later, DHA is reduced to AsA again by using GSH, which results in its oxidation to produce GSSG. Finally, this GSSG regenerates GSH by the activity of GR using NADPH as the electron donor (Figure 1). Both AsA and GSH are strong antioxidants, but the maintenance of their redox state is important in conferring stress tolerance in plants, which largely depends on the activities of the four enzymes that are associated with the AsA-GSH cycle [6,13]. In the next sections, we have described all of the components of the AsA-GSH pathway.

## 3. Components of AsA-GSH Pathway

### 3.1. Ascorbate

All living organisms either make AsA (also known as Vitamin C) or get it in their foodstuffs. Naturally abundant l-AsA is of a simplest chemical structure and is related to C6 sugars. It is a hexonic acid aldono-1,4-lactone (either l-galacturonic or l-gulonic acid), having an enediol group at C2 and C3 [14]. The enediol group enables l-AsA for donating one or two electrons to form an initial oxidized intermediate (MDHA) and further to an oxidized (DHA) form. The C5 and C6–OH group serves to provide alcoholic nature. They can react with produced acetals, ketals by reacting with aldehydes and ketones, respectively. Having two asymmetric C, l-AsA illustratesa positive optical rotation, which is unaltered by the acidicpH of solution but greatly affected by alkaline pH, which increases over +160° in 2N NaOH solution [14]. 

In solid-state, l-AsA is stable but oxidizes readily in solution, in particular in the presence of Cu, Fe, or alkali to form DHA. Afterward, two MDHA can undergo spontaneous reaction to rejuvenate one molecule of l-AsA and one molecule of DHA [15]. 

**Figure 2:**
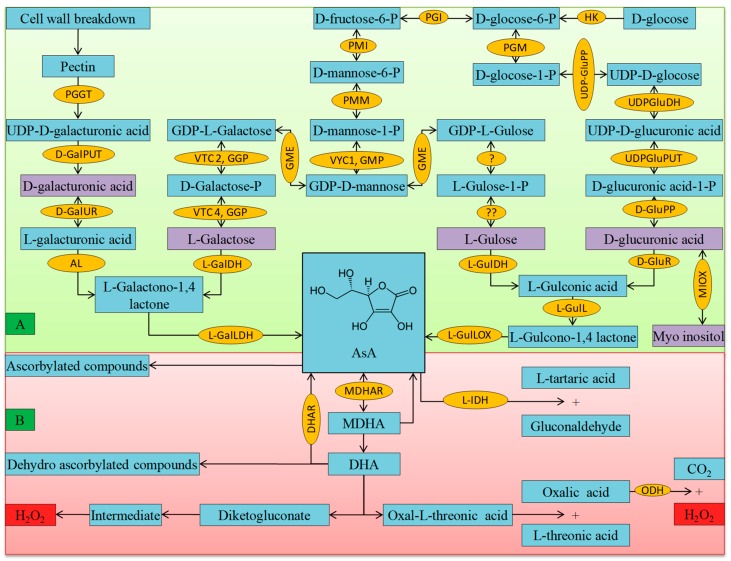
Ascorbate biosynthesis and metabolism is a complex set of reactions, some involving unidentified enzymes; some of the products are reactive and potentially damaging carbonyl compounds, (**A**) biosynthetic pathway; and, (**B**) regeneration and degradation pathways in plants. The metabolites in the violate box represent the name of each biosynthetic pathway. The elaborated name of enzymes are as follows (HK: Hexokinase; PGI: glucose-6-phosphate isomerase; PMI: mannose-6-phosphate isomeras; PMM: phosphomannomutase; TC1 or GMP: GDP-d-mannose pyrophosphorylase/mannose-1-phosphate guanylyltransferase; VTC2 or GGP: GDP-d-mannose 3′,5′-epimerase, GME: GDP-l-galactose phosphorylase;VTC4 or GPP:l-galactose-1-phosphate phosphatase; GalDH: l-galactose dehydrogenase; l-GalLDH: l-galactono-1,4-lactone dehydrogenase; ?: nucleotide pyrophosphatase or sugar-1-phosphate guanyltransferase; ??: sugar phosphatase; l-GulDH: l-gulose dehydrogenase; l-GulL: l-gulonolactonase; l-GulLOX: l-gulono-1,4-lactone oxidase; PPGT: polygalacturonate 4-alpha-galacturonosyltransferase; d-GalPUT: d-galacturonate-1-phosphate uridyltransferase; d-GalUR: d-galacturonate reductase; AL: aldonolactonase; PGM: phosphoglucomutase; UDPGluPP: UDP-glucose-pyrophosphorylase; UDP-GluDH: UDP-glucose dehydrogenase; UDP-GluPUT: glucuronate-1-phosphate uridylyltransferase; d-GluPP: d-glucurono-1-phosphate phosphatase; MIOX: myo-inositol oxygenase;d-GluR: d-glucuronate reductase; MDHAR: monodehydroascorbate reductase; DHAR: dehydroascorbate reductase;l-IDH: l-Idodonate dehydrogenase).

Ascorbate biosynthesis system is one of the ancient pathways and formed in very primitive life process on this planet. In plant tissue, AsA can be synthesized from several biochemical pathways. d-glucose is the primary substrate for producing AsA, and in this pathway, a set of ten reactions occurred (Figure 2). Ascorbate can be formed via four pathways viz. l-galactose, l-gulose, d-galacturonic acid, and myo-inositol pathway [16,17,18]. The biosynthesis of AsA is lineal with the cell wall formation. After the initial reactions, the d-galacturonic acid and L-galactose pathways both yielded l-galactono-1,4-lactone. Besides, in l-gulose and myo-inositol pathway, l-gulonic acid is produced, which is further hydrolyzes to form l-gulono-1,4-lactone, are catalyzed for the synthesizing of AsA in the mitochondria (Figure 2) [19].

In an organism, AsA metabolism comprises of biosynthesis (catabolism) and degradation (anabolism), and the balance between catabolism and anabolism determines the intracellular concentration of AsA. In the previous paragraph, we briefly discussed the biosynthesis of AsA. Hence, we will discuss the degradation and turnover of AsA in this paragraph. In some plants, the AsA turnover rate is relatively very high [20]. On the other hand, AsA is neither stable nor is restricted to oxido–reduction, which changes the equilibrium of AsA and DHA in plant tissue. The AsA pool undergoes turnover in plants. As AsA has prominent responsibility in the redox function metabolism, therefore the recycling of AsA from MDHA (catalyzed by MDHAR using NADPH) and DHA (DHAR using GSH) is the necessity to keep the redox balance as well as the higher total AsA pool [21]; and the functioning of the water-water cycle to optimize photosynthesis [22]. If the oxidized forms are not recovered, they will undergo further degradation to form oxalic or l-tartaric acids (Figure 2) [23]. In a plant cell, AsA act as a multifunctional biosynthetic precursor. While using radioactive ^14^C AsA, some studies tried to understand the degradation of AsA and DHA, but the mechanism is still not fully understood (Figure 2) [24]. However, it is well understood that the cleavage between C2 and C3 results in oxalate formation, whereas the cleavage between C3 to C6 produces l-threonate, via l-idonate [25,26]. Furthermore, DHA can be hydrolyzed into 2,3-l-diketogulonate, being further oxidized to unknown intermediate (Figure 2) and catalyzed by ascorbate oxidase (AO). Sometimes, this intermediate produces toxic H_2_O_2_ non-enzymatically and it may inhibit peroxidase [24,27].

### 3.2. Glutathione

Glutathione is an omnipresent low molecular weight tripeptide (γ-l-glutamyl-l-cysteinylglycine; γ-Glu-Cys-Gly), which is a strong antioxidant and an essential metabolite with a multifarious role in plants [28,29]. It was first discovered from yeast cells subsequently in many plants and animal tissue. Later on, in 1936, it was found as the reducing agent present in the plant tissue [30].

Although GSH is composed of glutamine (Glu), cysteine (Cys), and glycine (Gly), three essential amino acids; but some plant may contain homologues of GSH, where Gly is replaced by other amino acids [31]. In plants, reduced GSH accounts for >98% of total GSH [10]. Generally, cells possess three major reservoirs of GSH cytosol (80–85%), mitochondria (10–15%), and endoplasmic reticulum [32]. The thiol group is specific to γ-glutamyltranspeptidase (GGT) and it allows GSH a higher degree of stability [32,33]. Nevertheless, GSH plays a vital role, including antioxidant defense, xenobiotics detoxification, cell cycle regulation, and apoptosis, reserving cysteine, maintaining redox balance as well as immunity modulation and fibrogenesis [10,29].

**Figure 3:**
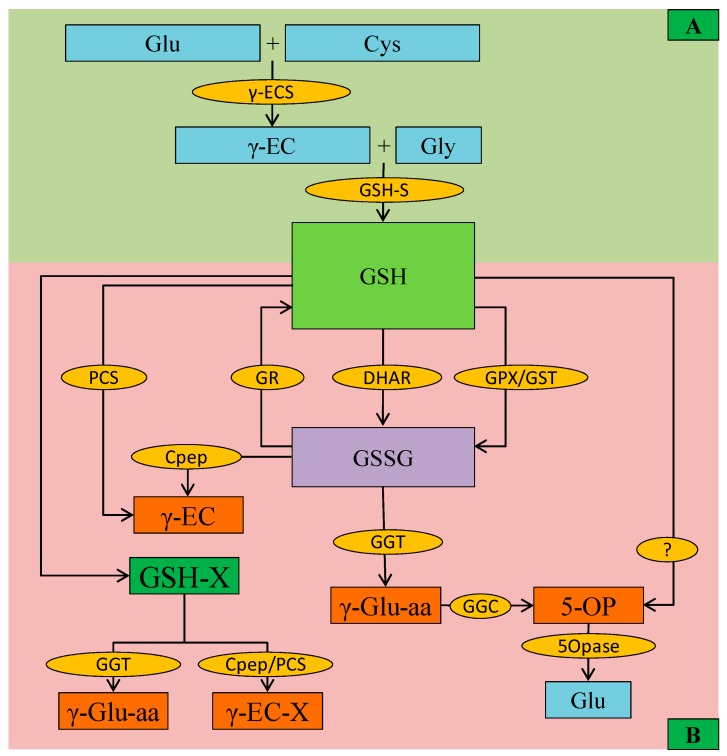
Glutathione biosynthesis, metabolism, and degradation in plants. (**A**) Biosynthesis the first step occurred in plastid: Glu and Cys form γ-glutamylcysteine (γ-EC) catalyzed by γ-EC synthetase (γ-ECS). The second step occurred in the cytosol or in plastid: γ-EC and Gly bond together to form GSH catalyzed by GSH-S (glutathione synthase). Further, GSH participates in ROS scavenging and is converted into GSH/glutathione disulfide (GSSG) by the enzyme glutathione peroxidase (GPX), glutathione *S*-transferase (GST), and DHAR. Further GSSG can be recycled to GSH by the activity of glutathione reductase (GR). (**B**) In the degradation pathway, GSH and S-conjugated compound (GS-X) can be degraded to γ-EC and γ-EC-X by phytochelatin synthase (PCS). While, carboxypeptidase (Cpep) and γ-glutamyl transpeptidase (GGT) both could degrade GS-X to form γ-Glu-aa (aa, amino acid) and γ-EC-X, respectively. Similarly, GSSG is degraded by GGT and Cpep to form γ-Glu-aa and γ-EC, respectively. Further, the produced γ-Glu-aa is converted to 5-oxoproline (5-OP) by γ-glutamyl cyclotransferase (GGC). Besides, GSH is also converted to 5-OP. Although it is thought that this reaction is catalyzed by GGC, still it is unclear. 5-OP is converted to Glu in the next step by the action of 5-oxoprolinase (OPase).

In plants, GSH biosynthesis involves two enzymatic steps, which require ATP and the constituent amino acids (Figure 3). In the earliest stratum, γ-glutamylcysteine (γ-EC) is produced by γ-glutamylcyteine synthetase (γ-ECS, EC 6.3.2.2) catalysis and participation from Glu and Cys. In the next stratum, GSH is synthesized from γ-EC and Gly via bonding from the Cys residue of γ-EC with α-amino group of Gly, catalyzed by GSH synthetase (GSH-S, EC 6.3.2.3, also known as GSH synthase). After synthesis in the cytoplasm, GSH is transported to other cellular organelles [34].

Glutathione is very important for various physiological processes, especially during abiotic stress; it coordinates with AsA turnover and is oxidized to GSSG [35]. Moreover, some other thiol-dependent enzymes, GPX and GST use GSH as co-factor, hence converted to GSSG, which is further reduced back to GSH, with GR catalysis. In higher plants, there are two genes that are reported to encode GRs (*GR1* and *GR2*), where *GR2* is essential for plant development [31,34].

The degradation of GSH is another important phenomenon of GSH metabolism (Figure 3). Up to now, as many as four types of GSH degrading enzymes have been described, which either use GSH or act on GSSG or other GSH-conjugates. Among them, carboxypeptidase activity could degrade GSH itself or GSH-conjugates. Cytosolic PCS is another enzyme that is responsible for the breakdown of GSH-conjugates that are mainly activated during metal/metalloid(s) stress. Another enzyme γ-glutamyl transpeptidase (GGT) acts in GSH transpeptidation or hydrolysis, which is further converted to free Glu by the action of GGC (γ-glutamyl cyclotransferases) and 5-oxoprolinase (5-OPase) (Figure 3) [31,36]. Moreover, another vacuolar GGTs have been reported in *Arabidopsis*, which breaks GSH-conjugates [37]; hence, along with PCS, GGTs is important for metabolizing GSH-conjugates that is formed during secondary metabolites synthesis [37,38].

### 3.3. Ascorbate Peroxidase

The class I heme-peroxidases; APX (EC 1.11.1.11) occurred in several isoforms in plant cell, viz. cytosolic APX isoform (cAPX), mitochondrial APX isoform (mitAPX), peroxisomal and glyoxysomal APX isoform (mAPX), and chloroplastic APX isoform (chAPX) differed in their substrate specificity, molecular weight, optimal pH ranges between 7 and 8 for maximum activity and stability [39,40,41]. More importantly, isoforms activity is not stable when AsA is absent. For example, AsA concentrations those are lower than 20 μM greatlyreduced chAPX activity. All of the APX isoforms are heme peroxidase they are inhibited by cyanide and azide. Iron plays a vital role in the APX catalytic site; hence, despite the presence of high AsA concentration, Fe deficiency reduced the activity of cAPX [42]. If the single Cys32 residue near arginine (Arg) 172 residue is altered, APX loses the ability to oxidize AsA to DHA, but it can oxidize other small aromatic molecules. Therefore, the APX properties differ with the guaiacol peroxidases, but they are 33% identical with cytochrome c peroxidase (CCP) [43]. 

During ROS (H_2_O_2_) detoxification, APX binds H_2_O_2_ producing intermediate (I), and heme iron [Fe(V)] is oxidized forming oxyferryl species (Fe^4+^ = O). Afterward, APX is regenerated from I, in a two-step reaction withAsA, where the AsA donate an electron and become oxidized. Detailed reactions are shown below (HS = Substrate, S = One electron oxidized form of the substrate).

APX + H_2_O_2_ → Intermediate (I) + H_2_O

Intermediate I + HS → Intermediate II + S

Intermediate II + HS → APX + S +H_2_O

**Figure 4:**
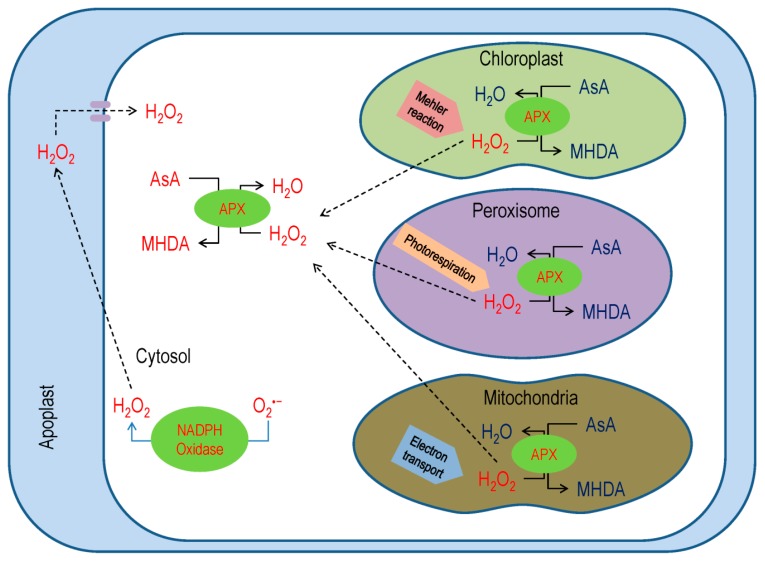
The function of Ascorbate peroxidase (APX) for the abolition of excess reactive oxygen species (ROS) generation in various cellular compartments. Additional details are in the text.

In plant cell, APX scavenges H_2_O_2_, which mainly participates in AsA-GSH cycle catalyzes the reactions produces MDHA (Figure 4) and, subsequently, MDHA yields DHA [6,10,44].

### 3.4. Monodehydroascorbate Reductase

**Figure 5:**
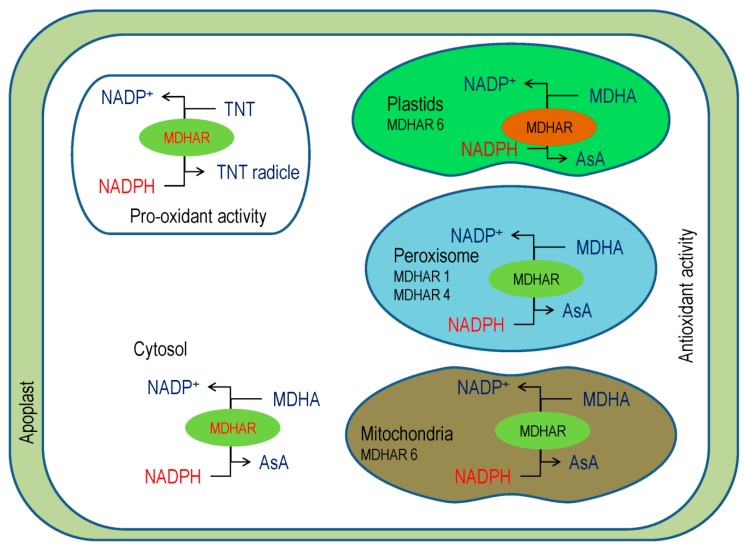
The antioxidant of MDHAR in regenerating AsA to support the removal of reactive oxygen species (ROS) (lower left) contrasts the pro-oxidant role of MDHAR creating 2,4,6-trinitrotoluene (TNT) toxicity.

The MDHAR (EC 1.6.5.4) helps in the revival of AsA [35], having several isoforms that are found in different organelles. Reports suggested that there are three MDHAR genes in tomato, five genes and six isoforms in *Arabidopsis* and rice, and as many as nineteen genes in wheat. In the plant cell, MDHAR activity was detected in different cell organelles, for instance, cytosol, mitochondria, chloroplasts, peroxisomes, and glyoxysomes (Figure 5) [45]. 

Although the enzyme is purified from several sources, the detailed structure of this enzyme was published in recent past. Begara-morales et al. [46] coined three-dimensional structure after conducting silico analysis of pea peroxisomal MDHAR. More recently, Park et al. [47] described details MDHAR composition from japonica rice. Those indicated that the structure of MDHAR consists of flavin adenine dinucleotide (FAD) and pyridine binding domain. The structure resembles Fe–S protein reductase [47]. The elucidated structure also indicates that rice MDHAR contains a typical α/β fold, and Arg320 and tyrosine (Tyr) 349 residues are vital for its activity. On the fad-binding domain, the fad is bounded by hydrogen and van der waals bond, where, Gly13, 15 and 297, alanine (Ala) 122 and 319, and threonine (Thr) 123 are involved and highly conservedin the bottom of the crevice. Moreover, Lys53 and Glu178 bridged together, which further bonded with proline (Pro) 49, where both Glu178 and Pro49, are highly conserved, as well as lysine (Lys) 53, Glu178, and FAD bonded each other. Among others, Arg48 compensate FAD phosphate group’s negative charge.

In rice, MDHAR α-helices surround β-sheets in the nicotinamide adenine dineucleotide (NAD)-binding domain, where the Tyr174, histidine (His) 315, and phenylalanine (Phe) 348 residues are shifted. Sandwiching between the FAD isoalloxazine ring and Tyr174, as well as steric hindrance moved Tyr174 away. The Phe348 residue shifts outward while His315 comes towards NAD binding site. In addition, the hydrogen bond is formed between Glu178 and nicotinamide ring; Arg202 and ribose and phosphate group, Glu314 and ribose; Glu196 and adenosine ring. Moreover, Glu196 offers rice MDHAR selectivity to NAD; preferring NADH over NADPH [47].

Interestingly, MDHAR can bind substrates other than MDHA, such as isoascorbic acid Evidence showed that phenoxyl radicals, like ferulic acid, quercetin, chlorogenic acid, and coniferyl alcohol, might be reduced by MDHAR [48]. In *Arabidopsis*, MDHAR activity reduces 2,4,6-trinitrotoluene (TNT) and creates its toxicity (Figure 5), but the MDHAR6 mutants are more tolerant, as TNT could not reduce and thus autooxidizes to creates O_2_^−^ [49].

Reports imply that MDHAR response to abiotic stress conditions by reducing MDHA that produces by the excess ROS scavenging, which was observed in many test species (Figure 5) [50,51]. The chlMDHAR is involved in photosynthetic activity during lack of peroxiredoxin [52]. In addition, chlMDAHR activity increased by three- to six-fold during pepper fruit ripening [53].

### 3.5. Dehydroascorbate Reductase

A major enzyme for GSH assisted DHA recycling is DHAR (EC 1.8.5.1), which is also known by GSH:DHA oxidoreductase or GSH dehydrogenase (AsA) [51,54]. This regeneration process is accomplished at alkaline pH and it is a well known biochemical reaction in plants.

The plant GSH-dependent DHAR is a monomeric enzyme, which is a member of the GSH*S*-transferase superfamily [55]. *Arabidopsis* possesses three functional DHAR encoding genes, DHAR1 (*At1g19570*), DHAR2 (*At1g75270*), and DHAR3 (*At5g16710*). In recent decades, the attention of researchers towards the DHAR activity in plants for regenerating DHA increased, and a number of investigations were carried out to elucidate the structure and molecular mechanism of DHAR. 

The overall three-dimensional structure of DHAR from different plant origin is almost identical, except with some additional short-chain before the α1-helix. The enzyme has several binding sites. The G site is responsible for binding the GSH in the enzyme. The GSH cysteinyl sulfur bonded Cys20 and occupied disulfide bond. The GSH γ-glutamyl is stabilized, via H-bonds H_2_O molecule, and then forms the backbone with serine (Ser) 73 and aspartic acid (Asp) 72. The Phe22 is engaged with the γ-glutamyl group by the van der Waals bond, in addition to hydrogen bonds with Lys59. The Val60 stabilizes the central cysteinyl region. The glycinyl group of GSH is loosely bound, forming a salt bridge with Lys47 [54]. 

The substrate-binding site or DHA binding site or H-site of DHAR enzyme typically exhibits more structural plasticity, but not simultaneously. From the structure of *Pennisetum glaucum* DHAR gene (*PgDHAR1)*, it was observed that Lys8 and Asp19 are responsible for DHA binding [56]. 

**Figure 6:**
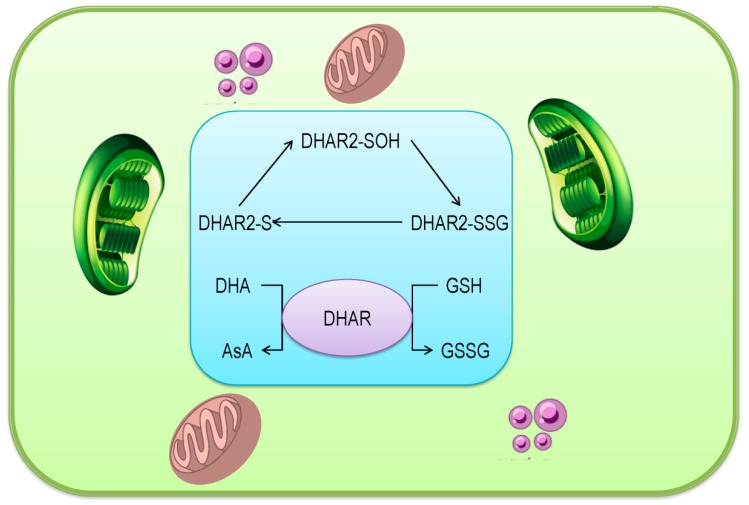
The mechanistic scheme, the ping-pong mechanism for the enzymatic reduction of dehydroascorbate (DHA).

The DHAR catalyzing accomplished by the following three reactions (Figure 6):

DHAR-S + DHA →DHAR-SOH + AsA (Reaction 1)

DHAR-SOH + GSH → DHAR-S-SG (Reaction 2)

DHAR-S-SG + GSH → DHAR-S + GSSG (Reaction 3)

Therefore, the process can be summarized by the following reaction:

2GSH + DHA → GSSG + AsA (Reaction 4)

As stated earlier, the integral function of DHAR is to reduce DHA to regenerate AsA. During this process, the active site of Cys is oxidized by DHA and further converted to the sulfenic acid. The reaction requires one molecule of H_2_O. Knockout mutants of *Arabidopsis DHAR1*, *DHAR2*, and *DHAR3*, did not show any significant differences in total AsA content until facing the abiotic stress condition, which confirmed the necessity of DHAR in reducing the DHA during stress [55].

### 3.6. Glutathione Reductase

The flavoprotein oxidoreductase GR (glutathione reductase, EC 1.8.1.7) and reduced GSSG to GSH, also known by the term GSR or NADP^+^ oxidoreductase, as it employs NADPH for its cellular activity. Although GR is stated as a dimer, the monomeric, heterodimeric, and heterotetrameric forms have also been illustrated [29].

No less than two genes that encoded GR were reported, viz. GR1 and GR2 from higher plants. Where GR1 is cytosolic or peroxisomal and shorter, contrary, GR2 comprises a long N-terminal sequence and mitochondrial or chloroplastic [57]. Up to date, several researchers reported GR isoforms in many plants, for instance, tobacco, spinach, etc. [10]. Although being found in above-stated organelles of the cell, the chloroplastic isoforms are accounted for 80% of GR [58]. The enzyme possesses a different quaternary structure that is based on the source from which it was purified [29]. 

Resembles with flavin-containing enzymes, GR exhibits the Rossmann folds, which is very much conserved and serves as the FAD and NADPH binding domains [59]. There is a controversy regarding the number of domain present in GR. Some reports suggested three, while some suggested four. Some researchers suggested an interface domain in GR protein; therefore, the enzyme has four domains, viz FAD-binding domain, NADPH-binding domain, GSSG-binding domain, and an interface domain [60]. Two Arg residues Arg287 and Arg293 are exclusively necessary for the enzymatic activity of GR [61]. Two Cys residue formed a disulfide bridge, which is redox-active and highly conserved. Serl64 replaces Cys residue in higher plants [62].

The enzyme shows high specificity to substrate binding, although the enzyme reduces GSH conjugates as well as mixed GSSG. Although Plant GR can employ NADP^+ −^, its affinity towards NADPH is high [29]. The catalytic mechanism of GR accomplishes in two steps. The first step involves NADPH dependent reduction of the flavin moiety, which is further oxidized, meanwhile the disulfide bridge in active site reduced to form an anion–thiolate and release Cys. In the next step, GSSG molecule binds in the active site forming a disulfide bond together with a Cys and histidine (His) separately of the active site. Afterward, one GSH leaves the His, while another followed it and then leaves the Cys residue leaving the disulfide the bridge in the enzyme active site [63]. 

**Figure 7:**
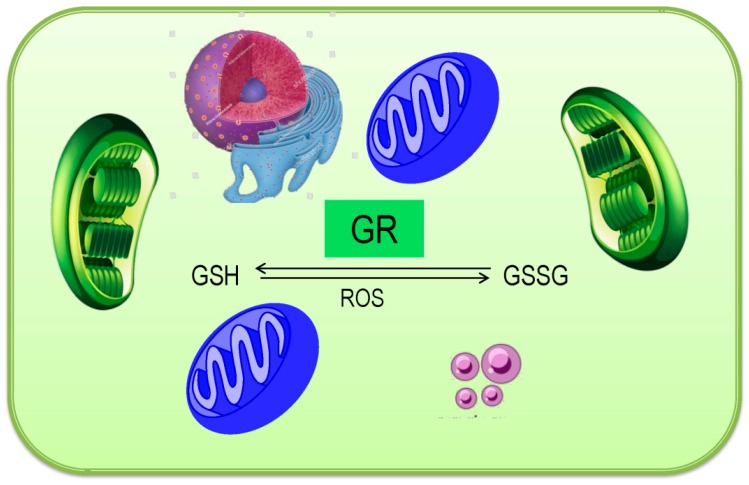
Mechanistic scheme for the enzymatic reduction of glutathione/oxidized glutathione (GSSG) in a plant cell.

The brief reaction catalyzing by GR is as follows (Figure 7):

GSSG + NADPH + H^+^ = GSH + NADP^+^

During catalysis, pH, and NADPH, and GSSG concentration modulate GR activity. It was reported that low NADPH concentration reduces the GR activity, while below pH 5.5 and over 7.0 is not suitable for GR actions. On the other hand, NADPH-induced GR inhibition was prevented by GSSG [64].

## 4. Ascorbate and glutathione Redox and its Role in Plant Metabolism

Balanced metabolism is the prerequisite for better productivity in plants, which is always disturbed due to biotic and abiotic stresses. Thus, redox balance is one of the key features of life, by which oxidized products are reduced for further oxidization and energy supply. Moreover, plant cells should counter the oxidation of vital cellular component that occurs continuously due to the presence of 21% atmospheric molecular O_2_, which is further complicated due to light-induced overproduction of ROS during photosynthesis. In addition, to keep the electron transport cascades active, simultaneous conversion of electron carriers between reduced and oxidized forms are required. Furthermore, photosynthetic and respiration needs regular electron flux to the electron transport chains from a different site. Therefore, the primary consequence is the generation of O_2_^−^and, subsequently, other ROS, from different enzyme catalysis [10,65]. Although playing a signaling role, over generation of ROS is harmful to cells; thus should be regulated to govern the redox homeostasis [66,67]. For example, AsA, GSH, tocopherols, thioredoxin, glutaredoxin, and peroxiredoxin, and energy metabolism mediators and electron carriers, for example, AsA/DHA, GSH/GSSG, FADH/FAD^+^, NADPH/NADP^+^, and NAD^+^/NADH play vital roles in plant cell for maintaining the redox balance and are termed as redox managers [68]. Among the redox managers, there are significant contributions drawn by AsA and GSH, hence in this section; we will discuss their role to keep redox balance as well as maintaining smooth cellular metabolism. 

Reports suggested that, under control condition, the AsA/DHA ratio remains >9. Ascorbate becomes oxidized during ROS scavenging, electron donation to photosystem II (PSII), violaxanthin de-epoxidation, and α-tocopherol reductive quenching [69,70]. While the direct reduction of MDHA by ferredoxin at photosystem I (PSI) and by MDHAR, as well as DHA reduction by GSH dependent DHAR activity, maintains a highly reduced state of AsA pool [2,71]. The biosynthesis and metabolism of AsA are discussed earlier in this article (Section 3.1). In the apoplast and vacuoles, AsA pool is an important redox buffer for ROS detoxification, where AsA recycling is mainly accomplished in the cytosol, and AsA/DHA acts as an oxidative stress sensor [72].

The GSH redox potential depends on the GSH concentration and the ratio of GSH/GSSG. In the GSH pool [GSH/GSSG], if the GSSG remains constant, but total GSH decreases, the equilibrium position dropped and redox balance is disrupted. Thus, proper judgment of the GSH/GSSG could give the idea of the redox ratio [73]. Glutathione serves in a multiplicity of metabolic functions; for instance, it participates in the regeneration of AsA from DHA in the chloroplast by DHAR [74]. Moreover, GSH plays a role in reducing glutaredoxins, functions as a precursor of phytochelatin (PC) synthesis for chelating heavy metal, signal transduction, sulfur metabolism, xenobiotics detoxification, and protects protein thiols against irreversible oxidation with disulfide formation or glutathionylation, which inhibited enzymes, like enolase and 6-phosphogluconolactonase [75]. Plant cells have distinct compartmentation of GSH. Although all other cellular compartments, except vacuole, contain GSH/GSSG redox buffer, the only vacuole is the storehouse of GSH where the GSH-conjugates are degraded [76]. 

As discussed in earlier (Section 2), both AsA and GSH are connected to the reactions network, the AsA-GSH pathway, and the cellular redox homeostasis depends on the pathway components [76]. In this cycle, AsA performs electron donation for APX that works for H_2_O_2_ detoxification. Due to its high attraction for H_2_O_2_, APX is capable of efficient ROS scavenging, even in a low concentration, which gives rise to DHA. The produced DHA is further recycled back, which maintains a high ratio of AsA/DHA. If DHA cannot reduce, it might further be irreversibly hydrolyzed, which decreases the ability of AsA redox pool [77]. In the catalysis process GSSG produced, which is further recycled back, which maintains not only a high GSH/GSSG ratio, but also the balance between GSH and AsA pools [10]. 

In addition, the redox couples of AsA/DHA and GSH/GSSG can also function in another way for accomplishing redox signaling [78]. As discussed earlier, the AsA/DHA couples create redox balance inside cells. Moreover, AO converts AsA to DHA in the apoplast [79], and it creates a redox gradient to connect intra- and extra-cellular atmosphere transverse the plasma membrane. Hence, AsA/DHA redox pair functions in apoplastic and cytoplasmic signals [80]. In contrast, the GSH/GSSG couple plays their functions in balancing intracellular redox potential, which in the intracellular redox signaling [81]. In this regard, the GSH distribution in different cellular organelle is very important for understanding cellular redox situation, for which signaling, as well as cellular metabolism, are smoothly going on [12].

## 5. Overview of Oxidative Stress and Antioxidant Defense in Plants

The production of ROS in living organisms is a usual cellular metabolism, and it is found in a large number in the internal constituents of the cell-like chloroplast, mitochondria, cytosol, peroxisomes, etc. [82,83,84].

**Figure 8:**
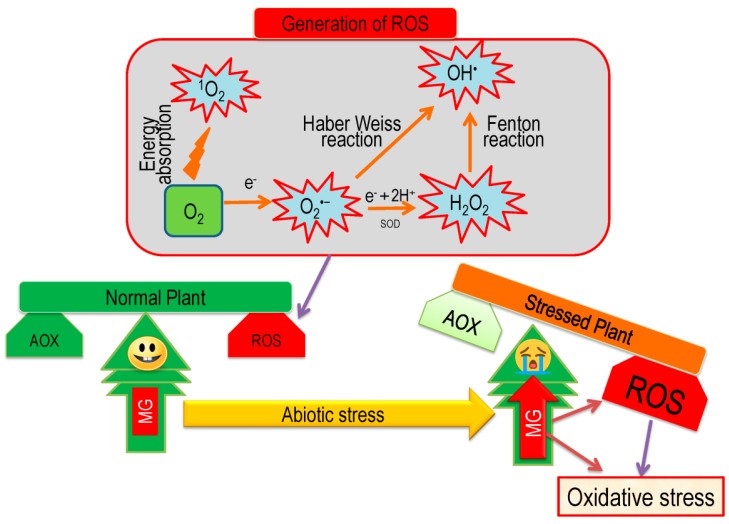
Abiotic stress-induced oxidative stress through the generation of ROS. Additional details are in the text.

Each plant cell maintains a dynamic balance between ROS and ROS-scavenging antioxidants. Abiotic stress destroys such cellular balance in favor of oxidative reactions by producing a huge amount of ROS [85]. Insufficient energy indulgence in the photosynthetic process during abiotic stresses reduces molecular oxygen and then produces a large amount of ROS, including H_2_O_2_, O_2_^−^, ^1^O_2_, OH^•^, and so on (Figure 8) [10,86]. Reactive oxygen species are extremely reactive molecules and they can damage a large variety of cellular biomolecules, including carbohydrates, nucleic acids, lipids, proteins, etc., and alter their functions [85,87]. In addition, MG, a cytotoxic compound and reactive oxidizer, spontaneously produced in a cell in little amount but under abiotic stresses, its production increased and participated in developing oxidative stress (Figure 8). Similar to ROS, MG production is increased under abiotic stress, which can damage the ultra-structural constituents of cell and cause mutation, and ultimately provokes programmed cell death (PCD) [10]. 

Besides causing oxidative stress, ROS and MG play signaling roles for stress tolerance, which controls acclimation and defense responses by modulating some antioxidants and their respective genes [10,86]. The excess generation of ROS and MG is also able to activate interruption in redox homeostasis, which can give the signal for cellular death or shortening plant life cycle [10,82].

**Figure 9:**
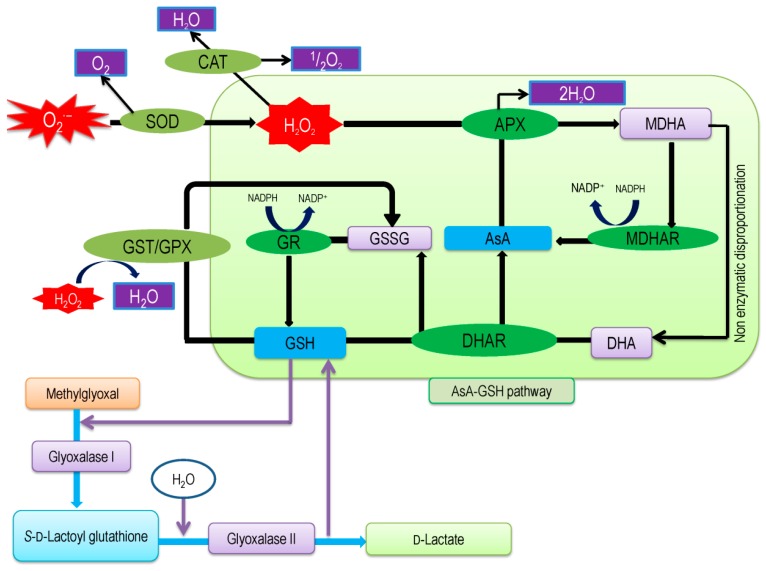
AsA-GSH pathway of the antioxidant defense system and its relation with the glyoxalase system. Additional details are in the text.

Plant cells have well established antioxidant defense and glyoxalase system for scavenging toxic ROS and MG, respectively. The antioxidant defense system consists of some non-enzymatic components (AsA, GSH, alkaloids, α-tocopherol, non-protein amino acids, and phenolic compounds) and enzymatic components (SOD, CAT, APX, MDHAR, DHAR, GR, GST, and GPX [5,28]). Within the antioxidant defense system, the AsA-GSH pool performs the direct and significant role for minimizing stress effect through scavenging of ROS by using key four enzymes, e.g. APX, MDHAR, DHAR, and GR [28,88,89]. In our previous section (Section 3.3, 3.4, 3.5, and 3.6), we elaborately discussed the function of these four enzymes in ROS detoxification. Usually, in the antioxidant defense system, SOD gives frontline protection against ROS by converting O_2_^−^ to H_2_O_2_. Subsequently, CAT and APX scavenge H_2_O_2_ to H_2_O. Glutathione peroxidase and GST also scavenge H_2_O_2_ to H_2_O with the help of GSH (Figure 9) [28].

Toxic MG is detoxified in the cell by glyoxalase system. Glutathione is not only the major element of AsA-GSH cycle, but it also plays a significant function in the MG detoxification system. Glyoxalase system is composed of two vital enzymes, Gly I and Gly II. In glyoxalase system, MG is detoxified to non-toxic compound in two steps reactions; in the initial step, MG is transformed to *S*-D-lactoyl-glutathione through the utilization of GSH and in the final step *S*-d-lactoyl-glutathione transformed in to d-Lactate, where GSH is recycled back [11]. Moreover, GSH contributes to metal chelation. It enhances the amount of PC under stress condition, which makes a complex with metal and drives into the cell vacuole as inert form [90].

## 6. Role of AsA-GSH in Regulating Oxidative Stress under Abiotic Stresses

Abiotic stress-induced excess ROS causes oxidative stress in plants followed by cellular damage, even death. Hence, the plant itself defends against this higher ROS accumulation by their defense mechanism. Plant significantly activates the AsA-GSH pathway for ROS detoxification. In this section, we will discuss the involvement of AsA-GSH cycle for alleviating oxidative stress upon various abiotic stresses reviewing recently published articles (Table 1, Table 2 and Table 3).

### 6.1. Salinity

One of the most devastating abiotic stress factors—salinity by which cultivable land is becoming barren thus reduces total crop production day by day. Oxidative stress is the most dangerous event under salt inundation is imposed by salinity-induced ionic and osmotic stress [10]. Hence, these ionic and osmotic stress both disturb the photosystem, and thus cause excess ROS, such as ^1^O_2_, O_2_^−^, H_2_O_2_, and OH. Salinity-persuaded acute ROS accumulations, then bother cellular redox followed by cellular damage counting membrane dysfunction, DNA damage, collapse the enzymatic action, along with distraction of the antioxidant defense system [91,92]. At this point, the plant synthesizes cellular AsA and GSH, which act as non-enzymatic antioxidants by involving their enzymatic components to detoxify ROS up to tolerable levels (Table 1).

However, the enzymes of AsA-GSH pathway showed their differential responses intolerant and sensitive varieties due to saline toxicity. Among salt-tolerant (Pokkali) and sensitive (BRRI dhan29) rice cultivars. Pokkali responded by enhancing the enzymatic activities of the AsA-GSH cycle, where, lowered APX and higher DHAR activity along with unchanged MDHAR and GR activities were found from BRRI dhan29. Rahman et al. [91,93] reported about the well involvement of AsA-GSH cycle in salt-stressed *O. sativa* where ROS generation was extreme. Here, salt exposed rice enhanced the reduced and oxidized GSH content with a lesser amount of AsA by the higher APX, MDHAR, DHAR, and GR activities against overproduced ROS. *Vigna radiata* was grown under the saline condition [94] and where salt-induced oxidative stress was marked with extreme O_2_^−^ and H_2_O_2_ overgeneration. Salt-stressed *V. radiata* augmented GSH and GSSG contents along with lowered AsA, whereas up-regulated the activity of all enzymatic antioxidants of AsA-GSH cycle and thus responded with elevated ROS [95]. Salt exposed *Lens culinaris* up-stimulated both MDHAR and DHAR activities, which resulted in a lesser amount of AsA and indicated the overproduced H_2_O_2_ detoxification [96]. Recently, Singh et al. [97] disclosed the incremental activity of enzymatic antioxidants, including APX, DHAR, and GR, with lower AsA, GSH, and GSSG contents, because of salt-induced higher ROS accumulation in *Solanum lycopersicum*. Similarly, 150 mM salt-treated *S. lycopersicum* also decreased AsA content, which might be used in H_2_O_2_ detoxification, while better GSH showed its role in lowering H_2_O_2_. Ahmad et al. [98] also observed higher APX, and GR activities, while MDHAR and DHAR activities again reduced as well as supported AsA-GSH mediated ROS regulation. Ahanger et al. [99] reported the same response of *S. lycopersicum* upon saline toxicity. Both activities of APX and GR were enhanced in salt-treated *Triticum aestivum* besides elevated H_2_O_2_ generation and resulted in higher GSH accumulation [100]. The activity of APX, MDHAR, DHAR, and GR enhanced in salt-stressed *S. lycopersicum* to check the excessive H_2_O_2_ generation, which resulted in lowered AsA and GSH contents [92].

The changes in AsA-GSH pathway were investigated in salt-stressed *Nitraria tangutorum* by applying a varied level of NaCl (100, 200, 300, and 400 mM) [101]. They noticed a gradual enhancement of AsA, DHA, GSH, and GSSG contents by keeping pace with sequential increment of salt-induced H_2_O_2_. Here, increased MDHAR and DHAR activities in stressed seedlings also contributed to increasing AsA, and higher DHAR and GR were responsible for better GSH and GSSG contents [92,102]. Talaat et al. corroborated these results with salt-exposed *Phaseolus vulgaris* [103]. Thus, as a part of plant antioxidant defense under salinity, AsA-GSH pathway is very efficient to regulate extra ROS for being tolerant.

### 6.2. Drought 

Drought is another most important abiotic stress, which generates excess ROS accumulation and thus causes variation in the enzymatic activities of AsA-GSH pathway for ROS detoxification. The enzymatic responses of AsA-GSH pathways varied, depending upon plant species, plant age, drought intensity, and duration [10]. Commonly, drought up-regulated the enzymatic antioxidant activities of AsA-GSH pool [10,104]. Plant tolerance to drought stress is categorized based on stress-induced endogenous antioxidants contents along with enzymatic activities (Table 2). *Dendranthema grandiflorum* responded differentially according to their tolerant and sensitive varieties, where tolerant one comparatively displayed better enzyme activity of antioxidants than sensitive [105]. Lou et al. [106] demonstrated how *T. aestivum* responded upon drought exposure. Hence, they noticed that the AsA-GSH cycle responded considerably with excess ROS generation by significant variation of GSH/GSSG and AsA/DHA redox along with the steady increment of H_2_O_2_. Their team also observed the enzymatic up-stimulation of AsA-GSH pathway to alleviate stress by scavenging excess ROS in *T. aestivum* spike. Thus, *T. aestivum* showed higher participation of AsA with higher APX activity in drought exposure for scavenging extra H_2_O_2_, as well as higher enzymatic activity to run the AsA-GSH pathway systematically [107].

Drought-stressed *A. thaliana* enhanced GSH and GSSG content along with the higher GR activity [108]. Hence, *Arabidopsis* showed the GSH dependent H_2_O_2_ detoxification to attain tolerance. Higher total AsA was accumulated in *Cajanus cajan* upon complete water restriction conditions for up to nine days to defend against excess H_2_O_2_ toxicity [109]. Hence, drought enhanced the enzymatic activity of APX, DHAR, and GR for decreasing GSH/GSSG, as well as controlling ROS level. 

Similarly, tolerant genotype VA13 of *Amaranthus tricolor* showed comparatively better tolerance under drought stress than sensitive one (VA15) by expressing differential responses of the enzymatic and non-enzymatic ROS detoxification pathways [110]. Hence, VA13 expressed remarkable increment in AsA-GSH redox by accelerating the enzymatic antioxidative actions by which increased non-enzymatic antioxidants (AsA and GSH) accumulation, which are vital for ROS detoxification. 

*Vigna radiata* responded differently regarding different drought intensity [111] to control diverse levels of ROS. Moderate drought imposed by 10% polyethylene glycol (PEG) induced comparatively lowered ROS than severe drought (by 20% PEG). Therefore, severe drought-stressed *Brassica* showed a larger use of AsA-GSH pathways against higher H_2_O_2_ generation than moderate stress. Here, higher stress caused a higher increase of APX activity along with lowest MDHAR and DHAR activity, while GR activity reduced differently than lower stress exposure to rapeseeds seedlings. Additionally, Hasanuzzaman et al. [52] also observed AsA and GSH both antioxidants contents reduced under severe drought condition, but increased under moderate stress. Bhuiyan et al. [104] found increased AsA content in *B. rapa* under drought (20% PEG). They also observed increased APX activity in drought-stressed seedlings, which assisted in efficiently scavenging the H_2_O_2_. Another two enzymes related to AsA regeneration MDHAR and DHAR also upregulated, as a result the AsA level was increased and strongly maintained its redox balance during oxidative stress situation. Nahar et al. [111] narrated the function of AsA as ROS detoxifier under drought stress where AsA content reduced in *V. radiata* with the increasing of ROS generation. Here, drought-induced higher APX activity enhanced the oxidation of AsA by scavenging H_2_O_2_ and improved GR activity increased the supply of GSH for involving ROS detoxification. *Anacardium occidentale* also showed the active participation of AsA-GSH cycle by integrative responses of both non-enzymatic and enzymatic antioxidants for drought-induced excess ROS regulation, where the higher accumulation of AsA and GSH, along with APX activity, coordinately reduced the overproduced H_2_O_2_ [112]. Thus, the AsA-GSH pathways involve in ROS detoxification as well as ROS homeostasis by eliminating excess ROS for keeping them up to the requirement of functioning cell signals.

### 6.3. Toxic Metals/Metalloids

Due to fast industrialization of the modern world and unrestrained anthropogenic activities, toxic metals/metalloids stresses have become a gargantuan problem for the plant growth and development [121]. Plants experience toxic metals/metalloids stress try to survive to some extent by using their well established antioxidant defense system. But, the activity and performance of defense system differ with stress concentration, stress duration, plant type, and age of the plant. 

The enzymes of AsA-GSH pathway confirmed their differential responses to different toxic metals/metalloids stress (Table 2). Mahmud et al. [122] confirmed that due to Cr stress, the few components of AsA-GSH pathway increased their amount or activity in *B. juncea* L. cv. BARI Sharisha-11. They found five days duration of 0.15 mM and 0.3 mM K_2_CrO_4_ treatment decreased the content of AsA, but did not change the GSH content. Moreover, activities of APX and GR were enhanced; however, the activities of MDHAR and DHAR were diminished. The higher APX and GR activity might play a function in scavenging excess ROS. A similar upregulation of APX and GR was also recorded in *B. napus* L. cv. BINA sharisha 3 due to Cd treatment [123]. From two separate experiments, they also found Cd stress (0.5 mM and 1.0 mM CdCl_2_) for 48 h decreased the AsA content, but increased GSH content only under 0.5 mM CdCl_2_ treatment. Exposure of *Gossypium* to 50 and 100 μM Pb(NO_3_)_2_ for six weeks increased the H_2_O_2_ content and APX activity [124]. The addition of 150 μM NiCl_2_·6H_2_O in growing media of *B. juncea* L. for one week increased the H_2_O_2_ content. Moreover, Ni stress decreased the AsA level but augmented the content of GSH and GSSG. Nickel also diminished the function of DHAR and MDHAR, however enhanced APX and GR activity [125]. Similar differential responses of AsA-GSH pathway components were also observed under As [126] and Al [50] toxicity. It can be stated that overproduced ROS plays the signaling role to some extent and inaugurate the higher activity of AsA-GSH enzymes under metals/metalloids toxicity. The upregulation of enzymes plays a significant role in maintaining the redox balance of AsA-GSH pathway under stress condition.

### 6.4. Extreme Temperature

Along with the rise in average global temperature, HT stress has been turned into a topic to be concerned about among environmentalists and researchers worldwide. In general, a 5 °C temperature rise above the optimum temperature of growth is considered to be extreme temperature stress or HT stress or heat shock to any plant species [142,143]. Heat stress causes denaturation of protein and membrane lipids, enzyme inactivation, inhibited protein synthesis, and loss of membrane integrity [144], which results from the disruption of cellular homeostasis through the ROS formed in a mass amount under heat stress [143,145]. Focusing on the role of AsA-GSH pathway to scavenge these ROS, different crop species under different levels of extreme or HT stress have been studied (Table 3).

Khanna-Chopra and Chauhan [146] selected a warmer season to induce HT stress to two different cultivars of wheat (*T. aestivum*), which are Hindi62 (heat-tolerant) and PBW343 (heat-sensitive). They sowed the wheat seeds in mid-January and considered it as heat stress environment, while the control plants were sown in mid-November and considered as the non-stress environment. Data were collected at seven days interval up to 35 days after anthesis (DAA), and the results showed a sharp increase in H_2_O_2_ content up to 14 days, but then declined. Whereas, MDHAR and DHAR enzymes’ activity only increased in Hindi62, but APX and GR activities showed a fluctuating pattern of alteration in both cultivars [146]. Another cereal *Z. mays* when experimented similarly with two different cultivars; LM-11 (heat-sensitive) and CML-32 (heat-tolerant), exposed to 40 °C for 72 h, resulted in higher APX and GR activities in CML-32 roots, while a reduction occurred in the shoot. In LM-11, none of the enzyme activity or AsA content was affected [147]. Higher levels of O_2_^−^ production rate and H_2_O_2_ content were observed in *Ficus concinna* seedlings under 48 h of HT (35 °C and 40 °C) stress condition, where AsA and GSH contents were unaffected at 35 °C, while declining AsA at 40 °C temperature [148]. The activity of APX, MDHAR, DHAR, and GR enzymes increased at 35 °C, but then again reduced at 40 °C to the level of control plants [148]. Under similar heat stress condition (40 °C, 48 h), *V. radiata* seedlings resulted in decreased GSH content and MDHAR-DHAR activities, but higher APX-GR activities [50]. Kiwi fruit (*Actinidia deliciosa*) seedlings, when exposed to 45 °C in an incubator for 8 h, resulted in higher AsA content and enhanced activity of all the AsA-GSH cycle enzymes [143]. Tomato seedlings were studied in two different aspects: short-term heat shock (40 °C, 9 h) [149] and long-term heat stress (38/28 °C day/night, seven days) [150]. In both experiments, the enhancement of O_2_^−^ generation rate and H_2_O_2_ content were recorded, but enzyme (APX and GR) activity was only increased at short-term stress condition [149], while the long-term heat exposure reduced all four enzymes activities and GSH content [150]. Similar enzymatic activity was observed in *Nicotiana tabacum* seedlings after seven days of heat (35 °C) stress [151]. From the above discussion, it can be stated that heat stress prevailing for longer duration is less likely to have the capability to modulate AsA-GSH pathway as compared to short-term heat stress.

### 6.5. Flooding

Changes in global climate result in the frequent or unexpected occurrence of heavy rainfall in different regions of the globe, which causes a sudden flood and disrupts the normal ecosystem [2]. Such changes in the ecosystem may cause the extinction of plants species and imbalance in the natural environment [2]. Flooding induced production of ROS and subsequent cellular damage has been authenticated in many studies so far [152,153,154]. Following are the discussion regarding crop species facing flooding stresses and modulation of their AsA-GSH pathway by flooding stress (Table 3).

Pigeon pea (*C. cajan*) seedlings that are exposed to waterlogged condition for six days revealed that tolerant cultivar could increase APX and GR activities, but a susceptible one cannot [155]. They also observed that, unlike other cases, waterlogging caused a lower accumulation of H_2_O_2_ and O_2_^−^ [155]. In another experiment with *V. radiata*, Sairam et al. [156] showed that waterlogging similarly reduced the H_2_O_2_ and O_2_^−^production rate in susceptible cultivar, while the tolerant ones remained unaffected. However, both APX and GR enzymes’ activity increased in tolerant genotypes, while the susceptible one got reduced [156]. The enhanced production rate of O_2_^−^and H_2_O_2_ content under flooding stress have been reported in cotton [154], Welsh onion [157], and clover [158] plants. Cotton (*G. hirsutum* cv. Siza) plants after three and six days of flood exposure raised the AsA content, but reduced the activity of APX, MDHAR, and GR [154]. A similar reduction in APX and GR enzymes activities was also recorded in Welsh onion (*Allium fistulosum* L.) after 10 days of waterlogging stress [157]. When *Z. mays* seedlings were waterlogged for 21 h at their root portions, they resulted in reduced AsA content and increased APX activity [159]. On the other hand, under long duration (14 days) flooding stress, *Glycine max* L. plants showed a reduction of GSH activity in roots and GR activity in the shoot, but the GSH in shoot and GR in root were not affected [153]. In case of complete submergence of *O. sativa* L. plants for two, four, or eight days, elevated levels GR enzyme activity was recorded, while APX enzyme activity increased only in tolerant cultivar [152]. Accordingly, the discussion reveals that the impact of flooding stress on AsA-GSH pathway varies depending upon the plant species and duration.

### 6.6. Atmospheric Pollutants

Atmospheric pollutants are the substances that are assembled in the air to a level or magnitude that is dangerous for living beings. Plants that are grown under different levels of atmospheric pollution have shown their oxidative stress responses and AsA-GSH pathway regulation in different manners (Table 3).

*Erythrina orientalis* plants were grown in three different locations of Philippines: La Mesa (a non-polluted area); and, Makati and Quezon (highly air-polluted cities). The results revealed that plants grown in the non-polluted area had lower activities of APX and GR as compared to the ones grown in highly polluted areas [160]. A similar increase in APX activity along with higher AsA content was recorded in *Prosopis juliflora* plants grown under polluted industrial region [161]. In a recent experiment, Lucas et al. [162] studied *Lolium perenne* plants that were grown under two different areas of Spain, Madrid, and Ciudad Real, where Madrid was considered to be more polluted than Ciudad Real. The findings indicated that the pollens of *L. perenne* accumulated higher concentration of H_2_O_2_ and in shoots APX and DHAR activity declined, but the activity of MDHAR and GR increased in the shoot of *L. perenne* plants that were grown in Madrid [162]. When rice seedlings were exposed to continuous O_3_ treatment, the results showed a remarkable increase in both O_2_^−^ generation rate and H_2_O_2_ content. In addition, contents of AsA and GSH reduced, while APX, MDHAR, DHAR, and GR activity increased upto 70 days of O_3_ exposure in SY63 cultivar and upto 79 days of O_3_ exposure in WXJ14 cultivar [163]. Ascorbate and GSH contents were not affected by O_3_ exposure in the *Populus* seedlings, but DHAR activity was lower, while the activity of GR and MDHAR was higher after 17 days of O_3_ treatment [164]. Young strawberry (*Fragaria* x *anansa*) seedlings were exposed to three different levels of CO, NO_x_, and SO_2_, which are as follows: CO @ 133, 267, and 533 ppm, NO_x_ and SO_2_ @ 25, 50, and 199 ppm corresponding to low, medium, and high dose, respectively. As a result of exposure to these atmospheric pollutants, H_2_O_2_ content as well as O_2_^−^ generation rate increased. However, at low and medium doses of their exposure APX and GR activity increased, while at a high dose that decreased [165]. All sorts of atmospheric pollutants have a remarkable effect on AsA-GSH pathway, but further studies are required to demonstrate that those pollutants completely induced the modification of the AsA-GSH pathway.

### 6.7. Other Stress

Conklin et al. confirmed the positive role of AsA in protecting plants from ultraviolet (UV) radiation [159], where they found that Vit-C deficient mutant of *A. thaliana* was suffered by stress-induced damages than that of wild type. AsA-deficient mutants also showed sensitivity to O_3_ stress due to a lower biosynthesis of AsA [171]. Gao and Zhang [172] reported that vitc1 mutants of *A. thaliana* showed physiological disorders and greater oxidative damages than the wild type, which was due to lower activities of antioxidant enzymes. Mutant plants also showed lower GSH/GSSG and higher DHA/(AsA+DHA) ratio than the wild type. Singh et al. [173] observed a decrease in AsA-GSH cycle enzymes in UV-exposed plants, which in turn affected the plants with oxidative stress. Similar to higher plants, marine macroalga *Ulva fasciata* also showed a positive correlation between enhanced the functions of AsA-GSH cycle and better tolerance of plants to UV radiation [174]. In their study, scavenging of H_2_O_2_ was regulated by AsA-GSH cycle components, especially APX and GR. Noshi et al. [175] reported that AsA-GSH redox pool provided better protection of *Arabidopsis* from high-light mediated oxidative stress, which was mainly attained due to the higher activities of DHAR. However, both AsA and GSH were found to be responsible for conferring high light (HL) stress [175]. Later, Zheng et al. [176] that susceptibility of *Arabidopsis* mutant was to HL stress were related to the deficiency of AsA and GSH. When AsA deficient *A. thaliana* mutant (vtc2-1) exposed to HL, they generated a high level of H_2_O_2_ (an oxidative stress marker) than the wild type, which was highly and negatively correlated with the total AsA content. The lack of AsA also resulted in lower chlorophyll (chl) content, chl fluorescence parameters, and PSII photochemistry [176]. Recently, Choudhury et al. [177] studied the metabolomics of *A. thaliana* grown under HL and found that the increased biosynthesis of GSH supports the photochemistry that supports *Arabidopsis* better survival under HL stress.

The pivotal role of the AsA-GSH cycle was also observed in low pH stress also. Bhuyan et al. [170] tested five spring wheat cultivars at different levels of low pH stress. Their observation exhibited that low-pH stress resulted in elevated O_2_^−^and H_2_O_2_ generation. A decrease in AsA content with increased DHA content was observed, although the APX activity decreased. Increased MDHAR activity was observed, but the ratio of AsA/DHA was not increased. Decreased GSH content and increased GSSG content were found where DHAR and GR activity decreased, resulting in a drop of the GSH/GSSG ratio.

## 7. Exogenous Use of AsA and GSH in Conferring Abiotic Stress Tolerance

While considering the vital role of both AsA and GSH and their redox researches have been trying to explore the possibilities of using exogenous AsA and GSH in protective plants from abiotic stress. However, the effects are not straightforward due to their species and dose dependency. In the next sections, we provided a summary of the recent results on plant abiotic stress tolerance while applying exogenous AsA and GSH.

### 7.1. Exogenous AsA

As a non-enzymatic antioxidant, AsA is vital for plant defense mechanism by involving in stress perception and subsequent signaling, and therefore plant responses [178]. Besides its regenerative nature, AsA is also able to donate electrons with and/or without the help of enzymes, and thus significantly detoxifies ROS [179]. Thus, exogenous AsA application is the most prominent for enhancing plant tolerance due to its efficient protection against lipids and proteins oxidation under abiotic stresses [180]. 

Ascorbate can be exogenously applied as a foliage application, seed treatment, and co-treatment for the alleviation of stress-induced damages [181]. Many researchers reported about the supplemental AsA-mediated antioxidant defense regulation in various plant species under different stressors, such as salt stress [182], drought [183], extreme temperature [184], ozone [185], and heavy metal stress [186].

Supplemental AsA application effectively lowered the oxidative stress in salt-stressed *Phaseolus vulgaris*, as indicated by the reduction of malondialdehyde (MDA) and ROS accumulation through activating their immune systems related with up-regulation of SOD, CAT and GR activities [187]. The AsA recovered salinity-induced oxidative damage in *Caralluma tuberculata* by lowering the activity of APX, POD, CAT, and GR, which were increased upon saline toxicity [188]. Exogenous AsA-induced plant tolerance, especially on the AsA-GSH pathway, is cultivar dependent [189]. Hence, they used both salt-tolerant *O. sativa* cv. Pokkali and salt-sensitive *O. sativa* cv. Peta to exogenously apply AsA as co-treatment with salinity and found a reduction of H_2_O_2_ generation in both cultivars. Here, AsA enhanced endogenous AsA and GSH, along with higher SOD, APX, and GR activities in salt-stressed both cultivars in line with lowered ROS and MDA production. However, Pokkali showed more prominent responses of salt tolerance than Peta. Finally, Wang et al. [189] suggested that exogenous AsA differentially increased the salt tolerance mechanism, and thus lessened salt-induced ROS in two rice cultivars. Exogenous AsA enhanced the salinity tolerance of *Z. mays* through protecting oxidative stress with stimulation of plant antioxidant defense [190]. In this study, AsA was used as seed priming against 100 mM NaCl, and AsA restored the salt-induced membrane damage. Hence, external AsA improved the non-enzymatic antioxidants, including Pro, AsA, and GSH accumulation, where SOD and GPX activities increased. Rady and Hemida [190] found lowered CAT activity in AsA treated seedlings under salt stress, which pointed out the AsA-induced decline of H_2_O_2_ generation.

Plants get relief from drought stress by exogenous AsA application, which was reported by previous researchers [114,191]. Alam et al. [114] studied the AsA induced attenuation of oxidative stress in *B. napus*, *B. campestris*, and *B. juncea* under 15% PEG, indicated by decreasing lipoxygenase (LOX) activity, H_2_O_2_, and MDA contents. This AsA mediated oxidative stress mitigation was described by AsA caused the strengthening of plant antioxidant defense mechanisms. Hence, exogenous AsA not only responsible for modulating AsA-GSH cycle, but also increased other enzymatic antioxidants activities, such as CAT, GPX, Gly I, and Gly II in all plant species, except GST, which was only increased in *B. napus*. Exogenous AsA mitigated the PEG-induced oxidative stress in *Z. mays* where AsA used as co-treatment, later endogenous AsA content increased, followed by scavenging surplus H_2_O_2_ generation and a reduction of lipid peroxidation [191].The higher transcript levels of SOD, CAT, APX, GR, MDHAR, and DHAR were induced in tall fescue by AsA application under PEG-induced water crisis, in respect with the only stressed condition [192]. Subsequently, Xu et al. [192] recommended AsA as a phytoprotectant to improved plants tolerances upon drought stress.

Exogenously applied 50 µM AsA decreased high temperature (HT, 45/35°C)-induced elevated H_2_O_2_ and MDA contents with lowered electrolyte leakage (EL) in *V. radiata* [184]. Hence, supplemental AsA altered the heat-induced lowered SOD, CAT, APX, and GR activities with increasing endogenous AsA and GSH contents.The AsA also enhanced the antioxidant capacity of tomato to cope with low-temperature stress [193]. The foliar application of AsA decreased the EL and MDA content in *T. aestivum* seedlings when exposed to the combined stress of herbicide and low temperature (−2 °C) [194]. This AsA-induced lowered oxidative damage might be because of ROS scavenging under stress indicated by AsA mediated lowered O_2_^−^ and H_2_O_2_, which were attributed by increasing POD, APX, and GR activities. 

Seed priming with AsA also increased plant tolerance to metal stress. Hence seed priming with AsA of *A. esculentus* showed the alleviation of Pb-induced oxidative stress that was confirmed by lowered H_2_O_2_ and MDA contents [135]. This AsA-induced alleviation of oxidative stress supported by exogenous AsA mediated increment of endogenous AsA contents, as well as upregulation of SOD, POD, and CAT activities in Pb-stressed *A. esculentus*. The AsA priming also increased the anthocyanins content in Pb-exposed seedlings, which again enhanced the metal tolerance by checking ROS production. Previously, exogenous foliar application of AsA on rice seedlings increased AsA, and GSH contents, while enhanced both AsA/DHA and GSH/GSSG redox status, along with higher APX and GR activities under Cd stress [195].

Alamri et al. [196] investigated the potentiality of exogenous AsA to remove the metal-induced oxidative stress in *T. aestivum*. They observed that AsA suppressed the higher content of MDA and H_2_O_2_ in Pb exposed seedlings by improving the antioxidant enzymes activities, including SOD, CAT, and GR. Thus, AsA-mediated higher activity of enzymatic antioxidants could be responsible for the lowered membrane damage indicated by EL as well as Pb tolerance.

The AsA supplementation also showed its effective role in the mitigation of Cd-induced oxidative stress. Zhang et al. [141] reported that AsA application, as a foliar spray, could become a potent tool to alleviate Cd toxicity in *Z. mays*. They used 0.1, 0.3, and 0.5 mM of AsA in against 3.36 mM Cd contamination, while they observed a remarkable gradual reduction of both H_2_O_2_ and MDA contents under stressed conditions with increasing AsA levels. Foliar AsA application improved endogenous GSH along with the augmentation of SOD, POD, CAT, and GR activities, which are in line with AsA-induced lessening of oxidative stress in Cd-exposed *Z. mays*. 

Thus, exogenous AsA application scavenges ROS in the plant under abiotic stresses, and then protects cell membrane stability. Therefore, reduced MDA content and EL were reported with AsA application as a sign of AsA-induced alleviation of oxidative damages. Accordingly, such exogenous AsA-induced strengthening of plant antioxidant defense, along with lessening oxidative stress, explained the potential of AsA for conferring abiotic stresses.

### 7.2. Exogenous GSH

At endogenous level, being an active participant of AsA-GSH cycle, GSH scavenges H_2_O_2_ in enzyme-dependent pathways; GSH is a substrate for GPX; GSH detoxifies lipid hydroperoxides together with GSTs; and, GSH/GSSG induces signals for abiotic stress adaptation [6]. Moreover, several research studies reported that the exogenous application of GSH proved to confirm the additional beneficial effects for enhanced the antioxidant defense system and abiotic stress tolerance. 

After exogenous GSH pretreatment, mung bean plants were imposed with HT (42 °C), and beneficial effects were noticed. It enhanced chl and leaf RWC; increased cellular GSH content and GSH/GSSG lowering GSSG content; amplified APX, MDHAR, DHAR, GR, GPX, GST, and CAT activities; exogenous GSH pretreatment upheld the activity of Gly I and Gly II of MG detoxification system. The upregulation of both antioxidant and glyoxalase system ensured the HT tolerance. Meanwhile, GSH supplementation with HT decreased H_2_O_2_, O_2_^−^, MDA level, the activity of LOX, and MG content [95]. Increased temperature (35 °C) in root-zone variably affected physiological processes, growth, and Calvin cycle, which mediated inconsistency in antioxidant components; HT also affected antioxidant enzymes’ gene expression of *Cucumis sativus* L. seedlings. HT-induced reduction of GSH content, the ratio of GSH/GSSG, photosynthetic pigments level, photosynthesis, and changes of linked gene expression were evident. HT also augmented soluble protein, proline (Pro), O_2_^−^ generation and MDA level, expression of genes, and antioxidant enzymes functioning. The application of supplemental GSH with HT upheld soluble protein, Pro, antioxidant enzymes activity, and its linked gene expression, as well as inhibited O_2_^−^ generation and lipid peroxidation, than to HT treatment without GSH [197].

Exogenous GSH improved AsA, and GSH contents, GSH/GSSG, APX, MDHAR, DHAR, and GPX activity of antioxidant system in drought exaggerated mung bean seedlings, which helped to relieve the adverse effect reducing the ROS including H_2_O_2_ and O_2_^−^, both in content and visually in the leaf spots of which were visualized through histochemical detection. Exogenous GSH also decreased the LOX activity, which caused the oxidation of lipid. Exogenous GSH also up-regulated the activity of Gly I and Gly II, therefore, reduce the toxic consequence of MG, and MG-induced oxidative damage [111].

Exogenous GSH (1.0 mM) positively regulated an antioxidant system in wheat plants facing lead (Pb) stress. The imposition of Pb diminished growth, the relative water content of leaf, and chl *a* and *b* content; amplified Pro level, H_2_O_2_, and O_2_^−^ generation, and lipid peroxidation. Glutathione supplementation with Pb stress improved the AsA and GSH contents, GSH/GSSG, activities of MDHAR, DHAR, GR, SOD, CAT, and GPX, and decreased oxidative damage. The decline of H_2_O_2_ and O_2_^−^ generation and membrane lipid peroxidation was clear evidence, together with an increased level of Pro and chl, which contributed overall tolerance to Pb toxicity [35]. Pretreatment with 100 μM GSH with 50 μM Cd reversed growth reduction and concealed Cd-provoked MDA buildup. In contrast to Cd compelled plants, GSH pretreatment reversed photosynthetic pigment destruction, downregulated Cd accumulation in root and shoot. Exogenous GSH considerably increased the functioning of POD and SOD. In contrast to the Cd affected plants, exogenous GSH pretreated plants extensively reassured decrease in Cu or augmented in Fe levels, which were due to Cd [198]. Exogenous GSH and Cys were applied on lead (100 and 500 mg L^−1^) affected *Iris lactea* var. chinensis, growth, accumulation of Pb, and nonprotein thiol (NPT) accumulation pattern were observed. The addition of GSH improved GSH biosynthesis in root and shoot. Endogenous shoot level Cys was recorded for exogenous Cys addition. Exogenous GSH application, together with buthionine sulfoximine (BSO) addition, regulated enzymes involved in GSH biosynthesis. This GSH played an imperative function in Pb accumulation and adaptation to this stress [199]. Exogenous GSH application improved the germination and growth of *Arabidopsis*, tobacco, and pepper under mercury (Hg) stress. Exogenous GSH also conferred Cd, Cu, and Zn stress tolerance. Exogenous GSH downregulated H_2_O_2_ and O_2_^−^ generation and MDA content, whereas upregulated chl level under Hg. Outstandingly, exogenous GSH reduced Hg accumulation in *Arabidopsis*. GSH showed high binding empathy to Hg, as compared to Cd, Cu, or Zn [200].

Salt-tolerant Pokkali and sensitive cultivar Peta of rice were scrutinized for the role of exogenous GSH on them. Exogenous GSH increased the activity of SOD, APX, and GR, the amount of AsA and GSH, and reversed chloroplasts’ H_2_O_2_ and MDA accumulation in either cultivar affected by salinity (200 mM NaCl). However, tolerance was prominent in cv. Pokkali [163]. The supplementation of GSH inverted the pessimistic properties of salinity stressed (NaCl 100 mM) tomato plants improving the transcript levels and activities of enzymes that are linked to GSH biosynthesis and metabolism. The biosynthesis-related enzymes were gamma-glutamylcysteine synthetase (γ-ECS), glutathione synthetase (GS), whereas, others were GST, GPX, and GR. Exogenous supplemental GSH helped to upregulate the activity of SOD, peroxidase (POD), CAT, APX, MDHAR, DHAR and GR, GSH level, and GSH/GSSG in salt-stressed plants [201]. Externally applied GSH lessened the oxidative damage in different soybean genotypes via reducing H_2_O_2_ and MDA level, which were produced due to salinity. Glutathione supplementation minimizing the oxidative damage further contributed in yield attributes, and yield performance, which was seeds plant^−1^ and pods plant^−1^, 100-seed weight and yield [202]. Defensive function of supplemental GSH (1 mM GSH) was examined for salt (200 mM NaCl) stressed mung bean. Mung bean plants when imposed with exogenous GSH and NaCl elevated AsA and GSH levels, GSH/GSSG, enhanced APX, MDHAR, DHAR, GR, SOD, CAT, GPX, and GST activities were recoded. Exogenous supplemental GSH also augmented the activity of Gly I and Gly II under salinity. Enhanced antioxidant and glyoxalase system components that resulted from the effect of exogenous GSH application had several beneficial effects. MDA, H_2_O_2_, and MG, O_2_^−^production turned down, and leaf RWC and chl level raise; all of which made mung bean seedlings capable to perform better under saline growing media [95]. Glutathione was exogenously applied on tomato plants affected by salinity. Additional of GSH decreased oxidative stress. The reason behind this was revealed as redistribution of light energy in PSII, higher cellular GSH, GSH/GSSH ratio and activities of SOD, CAT and APX, MDHAR, DHAR, GR, and GRx. Glutathione supplementation revolutionized growth inhibition, Na^+^and Cl^−^ions balance, and Na^+^/K^+^. Choloplast, as well as stomatal function related to photosynthetic performance, were documented to improve after the application of GSH with salinity [203].

## 8. Interaction of Other Pathways with AsA-GSH Pathways in Regulating ROS Metabolism

Beside oxidative stress mitigation, the AsA-GSH cycle also interacts other pathways to reduce ROS and oxidative stress. Therefore, in this section, we will discuss the potentiality of AsA-GSH pathway components and their interaction with other pathways to modulate the ROS metabolism in plants.

### 8.1. Interaction of AsA-GSH Cycle with NO Metabolic Pathway

Although AsA-GSH cycle protects cellular components from oxidative damage, its components, especially the proteins (APX, MDHAR, DHAR, and GR), are also vulnerable to the oxidative damage, which can modify their activity, hence breaking down the antioxidant defense. In plants, nitric oxide can be produced from several biochemical pathways, both oxidative and reductive [35]. In the GSH pool, the reduced form of GSH can interact with NO and produce GSNO, which is further catalyzed by the action of GSNO reductase (GSNOR) and release NO and GSSG, and maintains the equilibrium of NO and nitrosothiols, as well as balance the redox state in the cell [204]. Moreover, *S*-nitrosylation could modify protein interactions, thus tinkering the antioxidant response [205]. Reports suggest that all of the proteins of the AsA-GSH cycle are influenced through *S*-nitrosylation and/or nitration, which are accomplished from the interaction with NO. Among the AsA-GSH cycle, enzymes APX is the most studied, which is directly influenced by NO metabolism. For example, the inactivation of APX1 is caused due to the oxidation of Cys32 [205]. Contrary, nitrosylation of APX1 active-site Cys32 increases its activity and this post translation modification (PTM) is performed during salinity stress, which increases oxidative stress as well as *S*-nitrosothiols [206]. Among the other enzymes, MDHAR is negatively modulated by nitration, which cuses enzymatic inactivity by altering the position of the cofactor binding site [206], and hence disturb the AsA recycling process. Although information is available on the nitration and activity modulation of DHAR proteins [207,208], but the involvement of Try in this process, as well as the structural alteration impact of the enzyme, is still unclear. Moreover, GR is also targeted for nitration, which is reported to inhibit its activity in a mammalian cell, but the chloroplasic and cytosolic GR of pea is not affected by nitration [206]. 

### 8.2. Signaling Role of AsA-GSH Cycle Components and Interaction with Other Pathways

Ascorbate serves as the co-factor for redox enzymes, as well as a precursor for several biosynthetic pathways. In addition, AsA is an important reducing agent for Fe, Cu, and Mn, thus act as a pro-oxidant controlling toxic OH^•^ production from the Haber–Weiss and Fenton reaction [209]. Besides, its role as an antioxidant is the most important part of detoxifying ROS. As a pro-oxidant AsA regenerate α-tocopherol. Moreover, AsA also works in the photo-protection that is mediated by the xanthophylls cycle, where violaxanthin de-epoxidase use AsA as a co-factor [210]. Moreover, AsA is employed as the substrate for organic acid (oxalate and tartrate) biosynthesis (Figure 2). Rapid cell expansion is correlated with AO activity, which oxidized AsA [211]. During cell expansion, Pro residues present in the glycoproteins of cell wall undergo hydroxylation where prolyl hydroxylase use AsA as a cofactor [210]. Furthermore, AsA can potentially upregulate cytosolic free Ca^2+^ via anion channels and play a signaling role [212]. More than this breakdown of AsA to DHA by APX or AO creates an electrochemical gradient over the plasma membrane, which also has a signaling role.

Under abiotic stress conditions, GSH triggers adaptation or PCD by intercellular signaling [213]. Glutathionylation of protein Cys residues suggests its redox signaling role, which alters the transcription of proteins [214]. In *Arabidopsis*, stomatal movement induced from methyl jasmonate (MeJA) is regulated by intracellular GSH [215]. In tobacco, both GSH and GSSG application induce Ca^2+^ signaling as well as the expression of a specific gene, which supports the involvement of GSH with signal pathways that connect the Ca^2+^-dependent protein kinase [216]. The protein family peroxiredoxins (Prxs) are also GSH dependent and catalyze the reduction of H_2_O_2_ [217]. The GSSG can be exchanged with sulfhydryl groups of proteins and produce protein–GSH disulfide conjugates, which has a long half-life and plays a vital role in cellular signaling [218]. Moreover, GSH influences translation, and PTM of proteins, modulation of metabolism, and gene expression [219]. Hence, the mechanistic process of GSH signaling role should be focused on in future studies. 

### 8.3. AsA-GSH Cycle Interaction with Phytohormone Biosynthesis Pathways

Ascorbate regulates phytohormone biosynthesis; hence, modulating plant development [220]. Ascorbate shows activity, where cell developments are affected by hormonal signaling and modulate effective signaling processes [221]. The abscisic acid (ABA) involvement in stagnating growth and metabolism suggests the crucial role of AsA sensing for plant survival [222]. In addition, a number of dioxygenases that are directly related to hormonal biosynthesis require AsA is a cofactor [223]. Moreover, a low AsA induces PR proteins, but do not alter antioxidative enzymes. Thus, AsA acts as a “crosstalking” signal, where ABA acts as an important intermediary signal induces PR1 proteins in many plants. Hence, phytohormone signaling arises the AsA-dependent PR genes regulation [224]. On the other hand, 1-aminocyclopropane-1-carboxylate (ACC) synthase (*ACS*) and ACC oxidase (*ACO*) genesencoding ethylene biosynthetic enzymes is induced by GSH. Further, GSH increases serine acetyl transferase (SAT) level and confers Ni toxicity tolerance [225]. In rice, the overexpression of SA metabolism genes gave raise to both SA and GSH content under oxidative stress [226]. Therefore, GSH triggers phytohormones, and vice versa, along with other signaling genes [227].

### 8.4. Interaction of AsA-GSH Pathway with Glyoxalase Pathway

There is an intimate relationship between AsA-GSH cycle and the glyoxalase pathway through GSH, where it plays a vital role in the detoxification of MG. Methylglyoxal is a respiratory byproduct and produced usually in plants and detoxifies by the glyoxalase system. However, MG is overproduced under stress, which causes toxicity [51]. Moreover, MG can disfunctionate antioxidant enzymes [1]. In the MG detoxification process, Gly I (EC 4.4.1.5) and Gly II (EC 3.1.2.6) work simultaneously to detoxify MG (Discussed in Section 6). In this pathway, Gly I uses GSH as a cofactor and conjugates MG with GSH to form *S*-D-lactoylglutathione (SLG), Gly II, and then produce d-lactate breaking SLG, and regenerate GSH (Figure 9) [1], thus playing important interaction with glyoxalase system. 

### 8.5. Interaction AsA-GSH Pathway with Xenobiotics Detoxification Pathways

Xenobiotic detoxification involves the conjugation of toxic xenobiotics with GSH, which are further transferred to the vacuole by using ATP driven tonoplast transporter. This detoxification enables secondary metabolites biosynthesis as well as storing in the vacuole, such as anthocyanin. Plants are having GSH-dependent enzyme, GST, which detoxify herbicides by conjugating it with GSH. Therefore, the glutathionylated metabolites are imported to vacuolar by ABC (ATP-binding cassette) transporters. However, the GST mainly functions in catalyzing natural products that were observed with xenobiotics and, similar to those, catalyzes alternative GSH-dependent biotransformation reactions and binds and carries phytochemicals between cellular compartments [228]. 

### 8.6. AsA-GSH Cycle Interaction with Metal Chelation Process

Maintaining lower metal/metalloid(s) level inside the cell involves metal sequestration by low molecular weight thiols, for instance, metallothioneins (MTs) and phytochelatins (PCs). The two important enzymes involved in this process, glutaredoxin (GRx) and thioredoxin (TRx), are GSH dependent and neutralize H_2_O_2_ or controls of protein thiols [229]. On the other hand, PCs are another important chelating agent containing that thiol group that are upregulated by different metal/metalloid(s) [32]. The basic component for this PC is GSH. The biosynthesis of PCs is accomplished by the enzyme PC synthase (PCS), which requires GSH as a substrate. The enzyme is crucial for metal detoxification, metal homeostasis, and stress tolerance [137].

## 9. Genetic Manipulation of AsA-GSH Pathway and Its Role in Abiotic Stress Tolerance

The regulation of AsA and GSH pool plays an important role in mitigating oxidative stress in plants. To attain this, the regulation of the enzymes that are related to the AsA-GSH pathway is vital. There are many plant studies that considered the genetic manipulation of AsA-GSH pathway. These studied revealed that the overexpression of AsA-GSH pathway enzymes provided the plants better protection against oxidative stress under various environmental adversities (Table 4). Transgenic tobacco plants overexpressing *PcAPX* showed enhanced tolerance to salt and drought [230]. Transgenic plants exhibited a 347% increase in APX activities under drought stress, as compared to control, which resulted in a remarkable decrease in H_2_O_2_ content (136%) than that of wild type (309%). The ascorbate content was also higher 63%) when compared to wild type (42%). Similar results were also observed in the case of salt stress [230]. Chin et al. [231] found that transgenic *Arabidopsis* overexpressing *OgCytAPX1* scavenged ROS effectively and showed enhanced tolerance to salt and heat. The overexpression of *Malpighia glabraMDHAR* gene resulted in a higher biosynthesis of AsA, which provided tobacco plants tolerance to salt stress [232]. Shin et al. [233] observed that the coexpression of *B. rapaBrMDHAR* and *BrDHAR* genes provided a remarkable improvement of oxidative stress in *A. thaliana*. The overexpression of *BrMDHAR* and *BrDHAR* showed enhanced MDHAR and DHAR activities and higher AsA/DHA ration. These plants also provided better radical scavenging capacity, which resulted in lower H_2_O_2_ content. Yin et al. [234] found that *Arabidopsis* plants overexpressing the gene *AtGR1* conferred Al stress tolerance by reducing reactive carnoyl species, which was mainly due to higher GSH level and GR activity. The plant that overexpressed *AtGR1* also maintained the activity of H_2_O_2_-scavenging enzymes. For instance, GPX and APX activities in Al-treated plants were decreased by 21 and 46%, respectively, but the wild-type plants only showed 8 and 30% decreases in such activities [234]. 

Modulating several *NAC* genes [*NAC* domain consists of three different genes; *NAM* (no apical meristem)-*ATAF* (*Arabidopsis* transcription activation factor)-*CUC* (cup-shaped cotyledon)] are also an efficient way to transform the AsA-GSH cycle, consequently enhancing stress tolerance. The overexpression of wheat *TaNAC2* in *Arabidopsis* lines showed tolerance against freezing, salt, and drought stress by modulating the AsA-GSH cycle [235]. Moreover, ectopic expression of *SlNAC2* conferred both salt (200 mM) and drought (20% PEG) tolerance up to 10 days in transgenic *Arabidopsis* lines, which is correlated with the lower accumulation of ROS. In addition, the transcriptomic abundance GSH metabolizing genes was also observed in transgenic lines, leading to increased GSH synthesis and lesser oxidative damage [236]. 

## 10. Conclusions and Outlook

Ascorbate and GSH have roles in decreasing oxidative stress, and it has been reported in numbers of research findings. Most of the research findings reported about their roles in antioxidant defense system for scavenging ROS. However, exogenous GSH related research on antioxidant defense system needs further confirmation at the genetic and molecular level. Moreover, without the commonly known ROS, like H_2_O_2_, OH, O_2_^−^, etc. some other oxidative stress-inducing agents, like reactive nitrogen species, MG, etc., should be brought under consideration for research. How GSH can affect the generation of other kinds of oxidative stress-inducing damage. Research that is related to exogenous AsA or GSH-induced GST activity concerning xenobiotic detoxification is rare. The regulation of tocopherol by AsA or GSH can be an interesting area of research. For the reduction of metal-induced oxidative stress protection, GSH plays a vital role by producing PCs and inducing vacuolar sequestration. The credible function of AsA-GSH cycle in this area is so far to explicate. The GSH/GSSG redox is a well-reported term when discussing stress-induced oxidative damage and signal transduction process towards adaptation though the process is not well revealed. Interaction between and among the AsA-GSH cycle components and the hormones, other signaling molecules or any other molecules in oxidative stress, redox regulation, or plant adaptation process is not well understood. It is well known that chloroplast, its photosystem, and Calvin cycle activity or photosynthesis process is the maximum contributor of most of the ROS and oxidative stress under any abiotic stress condition. Several research findings reported about the role of AsA and GSH in improving the chl or carotenoid levels. However, very few of them reported regarding the roles of AsA and GSH in regulating stomatal conductance, Calvin cycle, RuBP activity/regeneration, or photosystem efficiency, which directly generates ROS and results in oxidative stress [203]. Some of the research findings show the positive roles of GSH improving/regulating Pro, which is cellular ROS scavenger or cytosol stabilizer to reduce ROS generation. These are the promising area of future research, which not only will alleviate the oxidative stress, but also improve the photosynthetic efficiency of plants for increasing plant production for the constantly growing population of the planet.

Although, in this review article, we focused on abiotic stress-induced oxidative damage and the role of AsA-GSH cycle to mitigate such adversities, biotic stress (fungi, bacteria, virus, nematodes, and parasitic organisms, etc.) might also alter the essential plant processes as well as cellular metabolism.For example, the production of ROS and oxidative stress, disruption of membranes, hampering photosynthesis, changing enzyme activities, cell death, and yield loss might also be attributed to biotic stress, which is in line with abiotic stress.Biotic stress also disrupts signal transduction, as well as transfigures signal pathways that are associated with stress acclimation. Over the past decade, AsA-GSH cycle has also emerged as an important component for the plant biotic stress response. Similar to abiotic stresses, biotic stresses also alters the metabolism and changes in antioxidant activity. Therefore, AsA-GSH cycle also directly impacts the important metabolomic processes, thus providing an important link between metabolism, signal transduction, and acclimation to plants during biotic stress.

## Figures and Tables

**Table 1:** Role of AsA-GSH in regulating oxidative stress under salinity and drought.

Plant Species	Stress Levels	Status of AsA-GSH Component(s)	ROS Regulation	References
*Triticum aestivum* L.	100 mM NaCl	GSH content increased by 15%; Stimulated APX and GR activities by 78% and 56%, respectively	Increased H_2_O_2_ content about 79%	[100]
*T. aestivum* L. cv. BARI Gom-21	12% PEG for 48 and 72 h	Decreased AsA content at 48 h, but after 72 h, AsA content again enhanced; Increased GSH and GSSG content where GSH/GSSG ratio decreased time-dependently; Enhanced the activities of APX, MDHAR, and GR	Enhanced the H_2_O_2_ content by 62% and increased O_2_^−^ accumulation	[113]
*T. aestivum* L.	10% PEG	Reduced AsA/DHA and GSH/GSSG redox; Increased enzymatic antioxidants actions of AsA-GSH cycle	Increased H_2_O_2_ production	[107]
*T. aestivum* L.	35–40% field capacity (FC) water	Increased GSH/GSSG by 64% while decreased AsA/DHA by 52% respective with a duration of stress; Enhanced APX, MDHAR, DHAR and GR activities	Increased H_2_O_2_ along with stress duration	[106]
*T. aestivum* cv. Pradip	150 and 300 mM NaCl	Reduced AsA content upto 52%; Increased reduced and oxidized GSH accumulation by 55% and 18%, respectively with 32% higher GSH/GSSG ratio; Increased APX activity with 29% reduction of GR activity; Slightly increased MDHAR and DHAR activity	Enhanced H_2_O_2_ generation by 60%	[28]
*Oryza sativa* L. cv. BRRI dhan47	150 mM NaCl	Increased GSH accumulation while reduced AsA content by 49% Increased GSH content and lowered the redox status of both AsA/DHA and GSH/GSSG; Upregulated the activity of APX, MDHAR, DHAR, and GR	Increased the production of O_2_^−^ with 82% higher H_2_O_2_ accumulation	[93]
*O. sativa* L. cv. BRRI dhan49	300 mM NaCl	Reduced AsA and GSH accumulation by 51% and 57%, respectively; Decrease GSH/GSSG redox by 87%; Showed lowered APX (27%), MDHAR (24%), DHAR and GR (25%) activities	Increased H_2_O_2_ content upto 69%	[114]
*O. sativa* L. cv. BRRI dhan54	300 mM NaCl	Improved AsA content by 51% with higher GSH content; Decreased GSH/GSSG ratio by 53%; Showed higher APX (27%) and DHAR activities while decreased both GR (23%) and MDHAR activities	Accumulated 63% higher H_2_O_2_ content	[114]
*Brassica napus* L. cv. BINA sharisha 3	100 and mM NaCl	Reduced the AsA content by 22%; Increased GSH content by 72% and GSSG content by 88%; Unaltered the GSH/GSSG ratio; Amplified APX activity by 32%, decreased DHAR activity by 17%; Slightly increased GR activity	Accumulated higher H_2_O_2_ content by 76%	[115]
*B. napus* L. cv. BINA sharisha 3	200 mM NaCl	Reduced the AsA content (40%) along with increased GSH (43%) and GSSG (136%) contents; Decreased the GSH/GSSG ratio (40%); Amplified the APX activity (39%) and reduced the MDHAR (29%) and DHAR (35%) activities; Improved GR activity (18%)	Showed 90% more H_2_O_2_ content	[115]
*B. napus* L.	15% PEG	The AsA accumulation remained unaltered and reduced the AsA/DHA ratio; Enhanced GSH content by 19% and GSSG by 67% and decreased GSH/GSSG ratio; Increased APX, MDHAR, DHAR and GR activities	Higher accumulation of H_2_O_2_ by 55%	[116]
*B. campestris* L.	15% PEG	Decreased AsA content by 27% with a decrease of AsA/DHA ratio; Increased GSH content by 33% with higher GSSG content by 79% and lowered GSH/GSSG ratio; Decreased DHAR activity	Higher accumulation of H_2_O_2_ about 109%	[116]
*B. juncea* L.	15% PEG	Increased the AsA content and did not affect the AsA/DHA ratio; Increased GSH content by 48% and GSSG by 83% and decreased GSH/GSSG ratio; Increased APX, MDHAR, DHAR and GR activities	Accumulation of 37% higher H_2_O_2_	[116]
*B. juncea* L. cv. BARI Sharisha 11	10% PEG	Reduced AsA content (14%) while increased both GSH (32%) and GSSG (48%) contents; Enhanced APX activity (24%); Decreased MDHAR and DHAR (33%) activities along with 31% increased GR activity	Acute generation of H_2_O_2_ (41%)	[117]
*B. juncea* L. cv. BARI Sharisha 11	20% PEG	Decreased AsA content by 34% while increased the content of GSH by 25% and GSSG by 101%; Up-regulated APX activity by 33%; Decreased activity of MDHAR and DHAR (30%)	Extreme generation of H_2_O_2_ by 95%	[117]
*B. napus* L. cv. BINA Sarisha 3	10% PEG	Increased AsA (21%), GSH (55%) and GSSG contents while decreased GSH/GSSG ratio Unaltered the activities of APX, and increased the activity of MDHAR, DHAR, and GR (26%)	Elevated the H_2_O_2_ production	[11]
*B. napus* L. cv. BINA Sarisha 3	20% PEG	Unaltered AsA content along with higher content of GSH (46%) and GSSG and reduced GSH/GSSG ratio; Reduced the APX and MDHAR activities along with the higher activity of DHAR and GR (23%)	Showed higher H_2_O_2_ production	[11]
*B. napus* L. cv. BINA sharisha 3	10% PEG	Increased AsA, GSH (31%) and GSSG (83%) accumulation with lowered GSH/GSSG ratio; Increased APX activity while reduced MDHAR and DHAR activities, but GR activity remained unaltered	Increased H_2_O_2_ content by 53%	[52]
*B. napus* L. cv. BINA Sharisha 3	20% PEG	Slightly increased AsA content with 26% and 225% increase of GSH and GSSG content, respectively; Reduced GSH/GSSG ratio; Increased APX activity while decreased the activity of MDHAR, DHAR, and GR (30%)	Increased about 93% H_2_O_2_ content	[52]
*B. rapa* L. cv. BARI Sharisha-15	20% PEG	Slightly increased AsA content with 72% and 178% increase of GSH and GSSG content, respectively; Reduced GSH/GSSG ratio by 38%; Increased APX, MDHAR, DHAR, and GR activity	Increased about 131% H_2_O_2_ content	[104]
*Cucumis melo* L. cv. Yipintianxia No. 208	50 mM of NaCl:Na_2_SO_4_:NaHCO_3_:Na_2_CO_3_ (1:9:9:1 M)	Improved AsA, GSSG and DHA contents; Lowered GSH content; Reduced the ratio of AsA/DHA and GSH/GSSG; Stimulated the activity of APX by 96% and DHAR by 38% while reducing the activity of MDHAR and GR by 48% and 34%, respectively	Increased H_2_O_2_ accumulation	[118]
*Solanum lycopersicum* L., var. Lakshmi	0.3 and 0.5 g NaCl kg^−1^ soil	Reduced AsA and AsA/DHA ratio; Lowered GSH and GSSG accumulation with decreased GSH/GSSG redox; Increased APX activity by 28%, DHAR activity by 28% and GR activity by 14%	Enhanced H_2_O_2_ and O_2_^−^ accumulation	[97]
*S. lycopersicum* L.cv. Boludo	60 mM NaCl, 30 days	Reduced the activities of APX, DHAR, and GR; Increased MDHAR activity	Higher H_2_O_2_ generation	[119]
*S. lycopersicum* L*. var.* Pusa Ruby	150 mM NaCl	Decreased AsA and GSH content with a higher content of DHA and GSSG; Increased APX, MDHAR, DHAr and GR activities	Higher generation of H_2_O_2_ and O_2_^−^	[92]
*S. lycopersicum* L. var. Pusa Rohini	150 mM NaCl	Reduced AsA content by 42%; Increased both GSH and GSSG accumulation; Enhanced the activity of APX and GR by 86% and 29%, respectively with reduction of the activity of MDHAR and DHAR by 38% and 32%, respectively	Accumulated about 3 fold higher H_2_O_2_ content	[99]
*S. lycopersicon* L. cv.K-21	150 mM NaCl	Reduced AsA content by 40% with 50% higher GSH content; Lowered GSSG content by 23% while increased GSH/GSSG ratio by 112%; Increased APX (86%) and GR (92%) activity along with the lowered activity of MDHAR (32%) and DHAR (30%)	Elevated H_2_O_2_ content about 175%	[98]
*Nitraria Tangutorum* Bobr.	100,200, 300 and 400 mM NaCl	Increased AsA, DHA, GSH and GSSG accumulation decreased their redox status; Enhanced the activity of APX and GR; Unvaried the activity of DHAR and MDHAR but increased DHAR activity only at 300 mM NaCl	Increased O_2_^−^and H_2_O_2_ content by 38–98 and 49–102% respectively	[101]
*Camellia sinensis* (L.) O.Kuntze	300 mM NaCl	Enhanced the AsA and GSH content; Increased APX activity	Elevated H_2_O_2_ and O_2_^−^ content	[120]
*Phaseolus vulgaris* L. cv. Nebraska	2.5 and 5.0 dS m^–1^ prepared from a mixture of NaCl, CaCl_2,_ and MgSO_4_	Increased AsA, GSH, DHA and GSSG accumulations; Enhanced AsA/DHA and GSH/GSSG status; Stimulated the enzymatic activity of APX, MDHAR, DHAR and GR activities	Accumulated higher H_2_O_2_ content	[103]
*Vigna radiate* L. cv. BINA moog-1	25% PEG	Reduced AsA content along with higher GSH content of 92%; Increased GSSG content by 236% and reduced GSH/GSSG ratio; Amplified the activity of APX (21%) and GR while reduced MDHAR and DHAR activities	Elevated H_2_O_2_ content by 114% with higher O_2_^−^ generation	[111]
*V. radiata* L.	200 mM NaCl	Reduced AsA content; Increased GSSG and GSH accumulation and lowered GSH/GSSG ratio; Amplified the activity of APX, MDHAR, DHAR, and GR	Increased H_2_O_2_ content by 80% and O_2_^−^ generation by 86%	[95]
*V. radiata* L. cv. BARI Mung-2	5% PEG	Reduced AsA content where decreased AsA/DHA ratio by 54%; Increased GSSG content; Upregulated the activity of APX and GR (42%) while downregulated the MDHAR (26%) and DHAR activities	Elevated H_2_O_2_ and O_2_^−^ accumulation	[50]
*Lens culinaris* Medik cv. BARI Lentil-7	20% PEG	Lowered AsA content with higher total GSH content; Unaltered the APX and GR activities while the increased activity of MDHAR and DHAR (64%)	Accumulated higher H_2_O_2_ content	[96]
*L. culinaris* Medik cv. BARI Lentil-7	100 mM NaCl	Reduced AsA content by 87% while increased total GSH content by 260%; Improved the activity of APX, MDHAR, DHAR (286%) and GR (162%)	Increased H_2_O_2_ content by 15%	[96]
*Anacardium occidentale* L.	21-day water withdrawal	Enhanced total AsA and GSH content; Increased APX activity	Reduced H_2_O_2_ generation	[112]
*Arabidopsis*	12-day water withhold	Showed higher GSH and GSSG accumulation; Reduced GSH/GSSG ratio; Increased GR activity	Increased H_2_O_2_ accumulation rate	[108]
*Cajanus cajan* L.	Complete water withholding for 3, 6 and 9 days	Decreased GSH/GSSG ratio; Increased the activity of APX, DHAR, and GR	Higher H_2_O_2_ content	[109]
*Amaranthus tricolor* L.cv. VA13	30% FC	Increased AsA and GSH contents by 286% and 98%, respectively; Improved APX, MDHAR, DHAR, and GR activity by 371%, 379%, 375%, and 375%, respectively	No increment of H_2_O_2_ content	[110]
*A. tricolor* L.cv. VA15	30% FC	Increased AsA and GSH contents along with higher redox status of AsA/total AsA and GSH/total GSH; Enhanced the activity of APX, MDHAR, DHAR, and GR by 37%, 45%, 40%, and 2%, respectively	Accumulated higher H_2_O_2_ content by 137%	[110]
*C. sinensis* (L.) O. Kuntze	20% PEG	Higher contents of both AsA and GSH; Enhanced the APX activity	Higher accumulation of H_2_O_2_ and O_2_^−^	[120]

**Table 2:** Status of AsA-GSH in regulating oxidative stress under metal/metalloid stress.

Plant Species	Stress Levels	Status of AsA-GSH Component(s)	ROS Regulation	References
*Brassica napus* L. cv. BINA sharisha 3	Cd (0.5 mM and 1.0 mM CdCl_2_), 48 h	Reduced AsA content by 20% under 0.5 mM and 32% under 1.0 mM CdCl_2_ treatment; Increased GSH content only under 0.5 mM CdCl_2_ stress but enhanced level of GSSG by 34% under 0.5 mM and 65% under 1.0 mM CdCl_2_ treatment; Increased function of APX by 39% and 43% under 0.5 mM and 1.0 mM CdCl_2_ treatment but MDHAR and DHAR activity were diminished in dose dependant fashion; GR activity increased by 66% due to 0.5 mM CdCl_2_ treatment but reduced by 24% due to 1.0 mM CdCl_2_ treatment	Enhanced H_2_O_2_ content by 37% under 0.5 mM and 60% under 1.0 mM CdCl_2_ treatment	[88]
*Gossypium spp.* (genotype MNH 886)	Pb [50 and 100 μM Pb(NO_3_)_2_], 6 weeks	Increased APX activity	Increased H_2_O_2_ content	[124]
*T. aestivum* L. cv. Pradip	As (0.25 and 0.5 mM Na_2_HAsO_4_7H_2_O), 72 h	Reduced AsA content by 14% under 0.25 and 34% underd 0.5 mM Na_2_HAsO_4_·7H_2_O treatment; Increased GSH content by 46% and 34%, GSSG content by 50 and 101% under 0.25 and 0.5 mM Na_2_HAsO_4_·7H_2_O stress; Enhanced APX function by 39% and 43% but decreased DHAR function by 33% and 30% under 0.25 and 0.5 mM Na_2_HAsO_4_·7H_2_O treatment; Increased GR function by 31% under 0.25 mM	Increased H_2_O_2_ content by 41% under 0.25 and 95% under 0.5 mM Na_2_HAsO_4_·7H_2_O treatment	[127]
*B. napus* L. viz. ZS 758, Zheda619, ZY 50 and Zheda 622	Cr (400 µM), 15 days	Increased GSH and GSSG content; Increased APX activity	Increased H_2_O_2_ content	[128]
*Oryza sativa* L. cv. BRRI dhan29	As (0.5 mM and 1 mM Na_2_HAsO_4_), 5 days	Decreased AsA content by 33 and 51% and increased DHA content by 27% and 40% under 0.5mM and 1mM Na_2_HAsO_4_ treatment, respectively; Decreased ratio of AsA/DHA; Enhanced GSH content by 48 and 82% under 0.5mM and 1mM Na_2_HAsO_4_ treatment, respectively; Enhanced GSSG content whereas lessened GSH/GSSG ratio by 25% under 0.5mM and 41% under 1mM Na_2_HAsO_4_ treatment; Augmented the function of APX, MDHAR, and GR, however, reduced the activity of DHAR	Increased H_2_O_2_ content by 65% and 89% under 0.5mM and 1mM Na_2_HAsO_4_ treatment, respectively	[126]
*O. sativa* L. cv. Disang (tolerant)	100 µM AlCl_3_, 48 h	Increased AsA content in both roots and shoots; Enhanced the GSH content in shoots; Higher activities of APX, MDHAR, DHAR, and GR,	Elevated the generation of H_2_O_2_ and O_2_^−^	[129]
*O. sativa* L. cv. Joymati (sensitive)	100 µM AlCl_3_, 48 h	Higher accumulation of AsA in both roots and shoots; Reduced the GSH content in roots while shoots content was unaltered; Increased APX, MDHAR, DHAR activities; Slightly increased GR activities	Higher accumulation of H_2_O_2_ and O_2_^−^	[129]
*V. radiata* L. cv. BARI Mung-2	Cd (mild: 1.0 mM CdCl_2_, severer: 1.5 mM CdCl_2_), 48 h	Declined AsA content by 31% due to mild and 41% due to severe stress; Enhanced DHA level and reduced AsA/DHA ratio; GSH content did not change due to mild stress but enhanced owing to stress severity; GSSG level enhanced, and GSH/GSSG ratio decreased in dose-dependent manner; Increased function of APX but lessened MDHAR and DHAR function due to both level of stress; GR activity increased only due to severe stress	H_2_O_2_ level and O_2_^−^ generation rate was augmented by 73% and 127% due to mild and 69% and 120% due to severe Cd stresses	[130]
*V. radiata* L. cv. BARI Mung-2	Cd (1.5 mM CdCl_2_), 48 h	AsA content decreased by 27%, and the ratio of AsA/DHA reduced by 80% whereas DHA content increased considerably; Augmented the function of APX and GR however lessened function of MDHAR and DHAR	Increased H_2_O_2_ level and O_2_^−^ generation rate	[131]
*O. sativa* L. cv. BRRI dhan29	Cd (0.25 mM and 0.5 mM CdCl_2_), 3 days	AsA content and AsA/DHA ratio reduced by 37% and 57% due to 0.25 mM CdCl_2_ and reduced by 51% and 68% due to 0.5 mM CdCl_2_, respectively; DHA content increased significantly; GSH content enhanced due to 0.25 mM CdCl_2_ stress, but reduced due to 0.5 mM CdCl_2_ stress; GSSG content enhanced by 76% under 0.25 mM and 108% under 0.5 mM CdCl_2_ stress; Reduced ratio of GSH/GSSG in dose dependant manner; Enhanced APX, MDHAR and GR activity	Enhenced H_2_O_2_ by 46% under 0.25 mM CdCl_2_ and 84% under 0.5 mM CdCl_2_ treatmen whereas O_2_^−^ generation rate increased in dose dependant manner	[132]
*O. sativa* L. cv. BRRI dhan29	Cd (0.3 mM CdCl_2_), 3 days	Lessened level of AsA and AsA/DHA ratio but enhanced DHA level; Enhanced the level of GSH and GSSG however lessened GSH/GSSG ratio; Enhanced the action of APX, MDHAR, and GR whereas declined DHAR function	Overproduced ROS (H_2_O_2_ and O_2_^−^)	[133]
*O. sativa* L. Zhunliangyou 608	Cd (5 μM Cd(NO_3_)_2_·4H_2_O), 6 days	Reduced AsA content; Increased GSH content; Slightly reduced the APX activity	H_2_O_2_ content increased by 22.73%	[134]
*Abelmoschus esculentus* L. Moench	Pb (100 mg L^−1^), 21 days	Increased AsA content	Enhanced H_2_O_2_ content	[135]
*B. juncea* L. cv. BARI Sharisha-11	Cr (mild: 0.15 mM K_2_CrO_4_, severe: 0.3 mM K_2_CrO_4_), 5 days	AsA content lessened by 19% due to mild and 32% due to severe stress whereas DHA level enhanced by 83% due to mild and 133% due to severe stress as well as AsA/DHA ratio lessened by 47% due to mild and 82% due to severe stress; GSH content did not change considerably but GSSG content enhanced by 42% due to mild and 67% due to severe stress as well as GSH/GSSG ratio lessened by 26% due to mild and 41% due to severe stress; The function of APX enhanced by 21% due to mild and 28% due to severe stress; The activity of MDHAR and DHAR reduced by 25 and 32% under mild and 31 and 50%, under severe stress, respectively; Mild stress increased the activity of GR by 19% while severe stress increased by 16%	H_2_O_2_ level enhanced by 24% and 46% due to mild and severe stress. Similarly, O_2_^−^ generation rate also raised in a dose-dependent manner	[122]
*B. campestris* L. cv. BARISharisha 9, *B. napus* L. cv. BARI Sharisha 13 and *B. juncea* L. cv. BARI Sharisha 16	Cd (mild: 0.25 mM CdCl_2_, severer: 0.5 mM CdCl_2_), 3 days	Decreased level ofAsA, augmented level of DHA as well as decreased AsA/DHA ratio in all studied cultivars; GSH and GSSG level enhanced, but GSH/GSSG ratio lessened in all studied cultivars; APX and GR activities of all species increased significantly under both levels of Cd toxicity	Enhanced H_2_O_2_ level and O_2_^−^ production rate in all tested cultivars in a concentration-dependent fashion	[136]
*B. juncea* L. BARI Sharisha-11	Cd (mild: 0.5 mM CdCl_2_, severer: 1.0 mM CdCl_2_), 3 days	Reduced AsA content with higher DHA content and thus decreased AsA/DHA ratio; Increased GSH and GSSG levels as well as declined GSH/GSSG ratio; APX activity increased where GR increased at mild stress but remained unaltered at severe stress; Decreased MDHAR and DHAR activities	Enhanced the H_2_O_2_ and O_2_^−^level	[137]
*V. radiata* L. cv. BARI Mung-2	Al (AlCl_3_, 0.5 mM), 48 and 72 h	Enhanced DHA content but reduced AsA level and AsA/DHA ratio; Increased level of GSH and GSSG but the diminished ratio of GSH/GSSG; Augmented APX activity but decreased MDHAR and DHAR activity	Enhanced H_2_O_2_ level by 83% and O_2_^−^ generation rate by 110%	[50]
*T. aestivum* L. cv. Pradip	Pb [mild: 0.5 mM Pb(NO_3_)_2_, severer: 1.0 mM Pb(NO_3_)_2_], 2 days	AsA decreased in a dose-dependent manner; Mild stress improved the GSH level, but severe stress reduced it; Increased GSSG content; Increased APX activity; Diminished activity of MDHAR and DHAR in a concentration-dependent fashion; Mild stress improved GR activity but severe stress reduced it	Mild stress increased H_2_O_2_ levels by 41%, but severe stress enhanced it by 95% while O_2_^−^ generation rate also increased in a dose-dependent manner	[35]
*B. juncea* L. cv. BARI Sharisha-11	Cd (mild: 0.5 mM CdCl_2_, severer: 1.0 mM CdCl_2_), 3 days	AsA content decreased by 24% due to mild and 42% due to severe stress whereas DHA level enhanced by 79% due mild and 200% due to severe stress; Decreased AsA/DHA ratio in dose-dependent manner; GSH and GSSG content enhanced by 19% and 44%, respectively, due to mild stress, while only GSSG content enhanced due to severe stress by 72%; The ratio of GSH/GSSG declined by 17% due to mild and 43% due to severe stress; Enhanced APX by 15% due to mild and 24% due to severe stress; The activity of MDHAR and DHAR reduced by 12% and 14% due to mild stress whereas 17% and 24%, due to severe stress, respectively; The activity of GR enhanced under mild stress by 16% and lessened under severe stress by 9%	Level of H_2_O_2_ enhanced by 43% due to mild and 54% due to severe stress. Augmented O_2_^−^ generation rate in a dose-dependent manner	[138]
*B. juncea* L. cv. varuna	Ni, (150 μM NiCl_2._6H_2_O), 1 week	AsA content decreased by 61% whereas GSH and GSSG content increased by 75% and 151%, respectively; Enhanced function of APX by 60% and GR by 72%; DHAR and MDHAR activities were decreased by 62% and 65%, respectively	Increased H_2_O_2_ by 3.23-fold	[125]
*Pisum sativum* L. cv. Corne de Bélier	Pb (500 mg PbCl_2_ kg^−1^), 28 days	Increased APX and GR activity	Increased H_2_O_2_ content	[139]
*O. sativa* L. cv. BRRI dhan54	Ni (0.25 mMand 0.5 mM NiSO_4_·7H_2_O)	Diminished content of AsA and enhanced content of DHA as well as the lessened ratio of AsA/DHA by 73% and 92% under 0.25 mM and 0.5 mM NiSO_4_·7H_2_O stress; GSH and GSSG level enhanced in a dose-dependent manner. However, the GSH/GSSG ratio reduced only under 0.5 mM NiSO_4_·7H_2_O treatment; Increased APX, MDHAR, DHAR and GR activity by 70%, 61%, 19% and 37% under 0.25 mM NiSO_4_·7H_2_O and 114%, 115%, 31% and 104% under 0.5 mM NiSO_4_·7H_2_O treatment, respectively	Increased H_2_O_2_ content by 28% and 35% due to 0.25 mM and 0.5 mM NiSO_4_·7H_2_O treatment	[68]
*Capsicum annuum* L.cv. Semerkand	Cd (0.1 mM CdCl_2_), 3 weeks	Enhanced AsA and GSH content	Increased H_2_O_2_ content	[140]
*C. annuum* L. cv. Semerkand	Pb (0.1 mM PbCl_2_), 3 weeks	Enhanced AsA and GSH content	Increased H_2_O_2_ content	[140]
*Zea mays* L. cv. Run Nong 35 and Wan Dan 13	Cd (50 mg 3CdSO_4_·8H_2_O kg^−1^ soil), 6 months	Decreased GSH content	Increased accumulation of H_2_O_2_	[141]

**Table 3:** Role of AsA-GSH in regulating oxidative stress under extreme temperature, flooding, and atmospheric pollutant.

Plant Species	Stress Levels	Status of AsA-GSH Component(s)	ROS Mitigation	References
*Actinidia deliciosa*	45 °C, 8 h	Increased content of AsA; Higher activity of APX, MDHAR, DHAR, and GR	Increased H_2_O_2_ content	[143]
*Zea mays* L. cv. Ludan No. 8	46 °C, 16 h	Decreased GSH, and GSSG content, but interestingly GSH/(GSH + GSSG) ratio increased; Reduced GR activity	-	[154]
*Cinnamonum camphora*	40 °C, 2 days	Reduced AsA content with higher DHA content; Increased GSH and GSSG content; Enhanced the activities of APX, MDHAr, DHAR, and GR	Higher content of H_2_O_2_ and O_2_^−^	[166]
*S. lycopersicum* L. cv. Ailsa Craig	40 °C, 9 h	Higher APX and GR activities by 74% and 45%, respectively	H_2_O_2_ content increased by 49%	[149]
*S. lycopersicum* L.cv. Boludo	35 °C, 30 days	Increased the APX, DHAR and GR activities; Reduced the MDHAR activity	Increased H_2_O_2_ content	[119]
*Vicia faba* L. cv. *C5*	42 °C, 48 h	Enhanced the AsA, GSH ans GSSG content significantly; The enzymatic activity of APX and GR also enhanced	Extreme accumulation of O_2_^−^ and H_2_O_2_	[167]
*V.radiata* L. cv. BARI Mung-2	40 °C, 48 h	Decreased 64% in AsA/DHA ratio; GSSG pool increased; Higher APX (42%) and GR (50%) activities but declined activities of MDHAR (17%) and DHAR	Higher H_2_O_2_ content and O_2_^−^production rate	[50]
*Z. mays* cv. CML-32 and LM-11	40 °C, 72 h	Increased AsA content in both shoot and root of tolerant (CML-32) one, but unaffected in the susceptible (LM-11) one; Both APX and GR activity increased in roots of CML-32 but reduced in the shoot	Higher H_2_O_2_ accumulation, especially in shoots	[147]
*L. esculentum* Mill. cv. Puhong 968	38/28 °C day/night, 7 days	AsA+DHA and DHA increased by 220% and 99% respectively; AsA/DHA ratio decreased by 33%.; Higher GSSG (25%), but reduced GSH content (23.4%) and GSH/GSSG ratio (39%); APX, MDHAR, DHAR and GR activities declined	Enhanced O_2_^−^ generation rate and H_2_O_2_ content by 129% and 33% respectively	[150]
*Nicotiana tabacum* cv. BY-2	35 °C, 7 days	Total GSH and AsA contents rose after 7 days heat stress; Increased MDHAR. DHAR and GR activities up to 72 h	The increasing trend of H_2_O_2_ generation was observed up to 72 h, and then a sharp decline occurred	[151]
*Ficus concinna var. subsessilis*	35 °C and 40 °C, 48 h	AsA content reduced at 40 °C but GSH content similar to control at both 35 and 40 °C; DHA content enhanced by 49% at 35 °C and by 70% at 40 °C; APX activity increased by 51% and 30% at 35 °C and 40 °C; Activities of MDHAR, DHAR, and GR increased at 35 °C, but GR activity decreased by 34% at 40 °C	At 35 °C, 103% higher H_2_O_2_ content and 58% higher O_2_^−^production rate and at 40 °C those were 3.3- and 2.2-fold respectively	[148]
*T. aestivum* cv. Hindi62 and PBW343	Heat stress environment, Late sown (Mid-January)	Higher activities of MDHAR and DHAR was observed in heat-tolerant (Hindi62) one whereas other enzyme activities seemed mostly to decline with time	The content of H_2_O_2_ was higher up to 14 DAA compared to non-stressed seedlings	[147]
*G. hirsutum* cv. Siza	Waterlogged pot for 3 days and 6 days	Increased content of AsA by 20% at 3 days and 30% at 6 days of waterlogging; Lower APX, MDHAR and GR activities	Enhanced O_2_^−^ generation rate by 22 and 53% and H_2_O_2_ content by 10 and 39% at 3 and 6 days of waterlogging, respectively	[154]
*Sesamum indicum* L. cv. BARI Til-4	Waterlogged pot by 2 cm standing water on the soil surface for 2, 4, 6 and 8 days	Reduced AsA content upto 38%; Enhanced GSH and GSSG content significantly; Increased APX and MDHAR activities; Reduced DHAR activity upto 59%; GR activity decreased upto 23%	Increased H_2_O_2_content sharply	[168]
*Z. mays* cv. Huzum-265 and Huzum-55	Root portions waterlogged for 21 h	Reduced AsA content in both cultivars; Increased APX activity in both cultivars	-	[159]
*Glycine max* L.	Waterlogged pot for 14 days	GSH activity declined sharply in roots but shoots unaffected; Reduced GR activity in shoots but roots unaffected	-	[153]
*Trifolium repens* L. cv. Rivendel and *T. pratense* L. cv. Raya	2 cm standing water on the soil surface for 14 days and 21 days	Increased contents of both oxidized and reduced AsA observed in both genotypes	Higher H_2_O_2_ generation in both genotypes	[158]
*V. radiata* L. cvs. T-44 and Pusa Baisakhi; and *V. luteola*	Pot filled with water to 1–2 cm height below the soil level, 8 days	Increased activities of both APX and GR in tolerant genotypes but in susceptible one, activities reduced	Reduced contents of O_2_^−^and H_2_O_2_ in susceptible (Pusa Baisakhi) cultivar	[156]
*O. sativa* L. MR219-4, MR219-9 and FR13A	Complete submergence for 4, 8 and 12 days	APX activity declined by 88% in FR13A under 4 days of submergence but decreased about 64 and 83% under 8 and 12 days of submergence; GR activity increased in FR13A and MR219-4 cultivars by 10- and 13-fold respectively after 8 days	-	[152]
*Allium fistulosum* L. cv. Erhan	Waterlogging (5 cm) at substrate surface for 10 days	Lower APX and GR activities	Increased rate of O_2_^−^ generation by 240.4% and 289.8% higher H_2_O_2_ content	[157]
*C. cajan* L. genotypes ICPL 84,023 and ICP 7035	Soil surface waterlogged (1–2 cm) for 6 days	Reduced APX and GR activities in susceptible genotype, which was higher in tolerant one	Lower accumulation of H_2_O_2_ and rate of O_2_^−^ generation	[155]
*S. melongena* L. cv. EG117 and EG203	Flooding with a water level of 5 cm, 72 h	Increased AsA content in susceptible EG117 genotype GSH content in both genotypes; Increased APX activity but decreased GR activity	-	[169]
*S. lycopersicum* cv. ASVEG and L4422	Flooding with a water level of 5 cm, 72 h	Increase in both AsA and GSH contents; Non-significant changes in APX and GR activities	-	[169]
*Lolium perenne*	Grown in an area with high air pollution	APX and DHAR activities decreased while MDHAR and GR activities increased	A higher concentration of H_2_O_2_ in pollens	[162]
*Populus deltoides* × *Populus nigra* cvs. Carpaccio and Robusta	O_3_ treatment (120 nmol mol^−1^ for 13 h), 17 days	No impact on AsA and GSH contents; DHAR activity decreased while GR and MDHAR activity increased	-	[164]
*Fragaria* x *anansa*	High dose of carbon monoxide (CO) nitroxide (NO_x_) and sulfur dioxide (SO_2_)	The activity of both APX and GR increased upto medium dose but reduced under high dose	H_2_O_2_ content as well as O_2_^−^ generation rate increased	[165]
*O. sativa* L. cvs. SY63 and WXJ14	Continuous O_3_ exposure for up to 79 days	Both AsA and GSH contents are more likely to decrease; APX, MDHAR, DHAR, and GR activity increased up to 70 days of O_3_ exposure	Both O_2_^−^ generation rate and H_2_O_2_ contents increased	[163]
*Prosopis juliflora*	Grown in the polluted industrial region	The content of AsA and APX activity increased under polluted environment	-	[161]
*Erythrina orientalis*	Grown in a polluted industrial area	Increased activities of both APX and GR enzymes recorded	-	[160]
*T. aestivum* L. cv. BARI Gom-26	Acidic pH (4.5) of growing media	Increased AsA and GSH content; Improved redox balance of GSH/GSSG; Increased activity of APX, MDHAR, DHR, and GR	H_2_O_2_ contents increased by 209%	[170]

**Table 4:** Overexpression of genes related to AsA-GSH pathway and their role in ROS scavenging.

Enzymes	Gene	Gene Source	Target Plants	Regulatory Effects	References
APX	*OsAPX2*	Rice	Alfalfa	Decreased H_2_O_2_ and MDA contents; Enhanced salt tolerance	[237]
APX	*PcAPX*	*Populus tomentosa*	Tobacco	Increased AsA content; NADP^+^/NADPH ratio; Decreased lipid peroxidation and H_2_O_2_ contents; Increased salt and drought stress tolerance	[230]
APX	*CaAPX*	*Camellia azalea*	Tobacco	Enhanced MDHAR and DHAR activity; Regulated ROS generation; Enhanced cold and HT tolerances	[238]
APX	*ScAPX6*	Sugarcane	*Nicotiana benthamiana*	Hormonal regulation; Lower ROS generation; Enhanced tolerance to Cu stress	[239]
APX	*OgCytAPX1*	*Oncidium*	*Arabidopsis*	Efficient ROS scavenging capacity; Maintained redox homeostasis and increased GPX activities which resulted in lower H_2_O_2_	[240]
MDHAR	*Am-MDAR*	*Avicennia marina*	*A. marina*	Increase in MDHAR and GR activity; Increased AsA content; Decreased lipid peroxidation; Improved salt-induced oxidative stress	[241]
MDHAR	*MgMDHAR*	*Malpighia glabra*	Tobacco	DHAR activity increased by 1.8–2.1 fold; AsA/DHA increased by 81–84%; Lipid peroxidation decreased by 41–62%	[232]
MDHAR	*BrMDHAR*	Rapeseed	*Arabidopsis*	AsA/DHA ratio increased by 7%; Decrease of H_2_O_2_ content by 55%; Radical scavenging was 16% higher	[233]
DHAR	*DHAR*	Human	Tobacco	No changes in AsA content; Enhanced tolerance to chilling and salt stress	[242]
DHAR	*DHAR1*	Rice	*Arabidopsis*	AsA content increased by 1.4 fold; Enhanced tolerance to salt	[243]
DHAR	*DHAR*	Human	Tobacco	Increase in AsA content; Increased the activities of SOD and APX; Enhanced salt tolerance	[244]
DHAR	*DHAR*	*Arabidopsis*	*Arabidopsis*	AsA content increased by 2.0–4.25 fold; Enhanced tolerance to HT stress	[245]
DHAR	*DHAR*	Rice	Tobacco	AsA content increased by 1.6-fold; Improved tolerance to salinity and chilling	[246]
DHAR	*Br* *DHAR*	Rapeseed	*Arabidopsis*	AsA/DHA ratio increased by 11%; H_2_O_2_ content decreased by 56%; Radical scavenging was 16% higher	[233]
GR	*AtGR1*	*Arabidopsis*	*Arabidopsis*	Enhanced GSH content and GR activity (0.4- to 1.0-fold higher); H_2_O_2_ content reduced by 26%	{234]
